# Rem2 stabilizes intrinsic excitability and spontaneous firing in visual circuits

**DOI:** 10.7554/eLife.33092

**Published:** 2018-05-29

**Authors:** Anna R Moore, Sarah E Richards, Katelyn Kenny, Leandro Royer, Urann Chan, Kelly Flavahan, Stephen D Van Hooser, Suzanne Paradis

**Affiliations:** 1 Department of BiologyBrandeis UniversityWalthamUnited States; 2 Volen Center for Complex SystemsBrandeis UniversityWalthamUnited States; 3 National Center for Behavioral GenomicsBrandeis UniversityWalthamUnited States; RIKENJapan

**Keywords:** intrinsic excitability, Rem2, plasticity, activity-dependent, homeostasis, Mouse

## Abstract

Sensory experience plays an important role in shaping neural circuitry by affecting the synaptic connectivity and intrinsic properties of individual neurons. Identifying the molecular players responsible for converting external stimuli into altered neuronal output remains a crucial step in understanding experience-dependent plasticity and circuit function. Here, we investigate the role of the activity-regulated, non-canonical Ras-like GTPase Rem2 in visual circuit plasticity. We demonstrate that *Rem2^-/-^* mice fail to exhibit normal ocular dominance plasticity during the critical period. At the cellular level, our data establish a cell-autonomous role for Rem2 in regulating intrinsic excitability of layer 2/3 pyramidal neurons, prior to changes in synaptic function. Consistent with these findings, both in vitro and in vivo recordings reveal increased spontaneous firing rates in the absence of Rem2. Taken together, our data demonstrate that Rem2 is a key molecule that regulates neuronal excitability and circuit function in the context of changing sensory experience.

## eLife digest

Throughout our waking hours, we experience an ever-changing stream of input from our senses. The brain responds to this varying input by adjusting its own activity levels and even its own structure. It does this by changing the strength of the connections between neurons, or the properties of the neurons themselves. Known as plasticity, this process of continuous change enables the brain to develop, learn and to recover from injury.

The visual systems of mammals are particularly well suited to studying how sensory experience alters the brain. Studies in animals show that lack of sensory input to one or both eyes during a critical period in development causes long-lasting changes in the brain’s visual circuits. Similarly, children with the condition amblyopia or ‘lazy eye’ – in which one eye has impaired vision and the brain ignores input from that eye – can end up with permanent deficits in their vision if the condition is not treated during childhood. Changes in sensory input are thought to trigger plasticity in the brain by altering the activity of specific genes. But exactly how this process works is unclear.

Moore, Richards et al. now show that a gene called *Rem2* has an important role in regulating visual plasticity. In the key experiments, young mice had their vision in one eye blocked for a few days. Analysis of their brains showed that mice that had been genetically modified to lack the *Rem2* gene responded differently to this change in their environment (i.e. the loss of input to one eye) than their normal counterparts. Further experiments suggest that *Rem2* regulates the excitability of individual neurons: that is, how much the neurons respond to any given input. In the absence of *Rem2*, neurons in visual areas of the brain become hyperactive. This prevents them from adjusting their activity levels in response to changes in sensory input, which in turn leads to impaired plasticity.

Being able to harness the brain’s visual plasticity mechanisms on demand, for example by regulating *Rem2* activity, could benefit individuals with disorders such as amblyopia. *Rem2* is also active in many other parts of the brain besides those that support vision. This suggests that manipulating this gene could affect numerous forms of plasticity. However, various barriers must be overcome before we could use this approach to treat brain disorders. These include obtaining a more in depth understanding of the role of the *Rem2* gene in the human brain.

## Introduction

Ocular dominance (OD) plasticity in the mammalian visual system, induced by monocular eyelid suture, is one of the best-studied models of the influence of experience on neural circuit development. The current consensus view holds that OD plasticity is achieved through an ‘early-phase’ decrease in responsiveness to the deprived, contralateral eye through Hebbian-like LTD mechanisms (Cooke and Bear, 2010; Crozier et al., 2007; Kirkwood et al., 1996; Rittenhouse et al., 1999; Yoon et al., 2009), followed by a ‘late-phase’ homeostatically-regulated increase in responsiveness to the open, ipsilateral eye through synaptic scaling up and increases in intrinsic excitability (Kaneko et al., 2008b; Lambo and Turrigiano, 2013; Mrsic-Flogel et al., 2007; Smith et al., 2009). While significant attention has been given to the role of synaptic plasticity mechanisms in regulating experience-dependent changes in circuit function (Desai et al., 2002; Maffei and Turrigiano, 2008; Turrigiano et al., 1998), far less is known about the contribution of altered intrinsic neuronal properties to this process.

From crustaceans to mammals, neurons respond to decreased activity by modulating their ionic conductances in order to change their intrinsic excitability and re-establish an appropriate firing rate (Aizenman et al., 2003; Desai et al., 1999; Liu et al., 1998; Marder and Prinz, 2003; Pratt and Aizenman, 2007; Siegel et al., 1994). Underlying this homeostatic regulation of intrinsic excitability are activity-dependent changes in gene expression (Dong et al., 2006; Turrigiano, 2008) and recent modeling studies illustrate that the rate of calcium-dependent gene transcription serves as a central point of regulation underlying changes in intrinsic excitability and neuronal conductances (O'Leary et al., 2014). Interestingly, this model posits the existence of a key regulator or ‘sensor’ molecule whose main function is to assess cell-wide activity levels, indicated by changes in Ca^2+^ influx, and implement downstream signaling mechanisms that alter intrinsic excitability (Liu et al., 1998; O'Leary et al., 2014; Siegel et al., 1994). However, the identity of such sensor molecules and signaling pathways remains largely unknown.

The activity-regulated Ras-like GTPase Rem2 is an excellent candidate to link the activity of a neural circuit with functional plasticity. Rem2 is a member of the Rad/Rem/Rem2/Gem/Kir (RGK) protein family, a Ras-related subfamily of small GTPases (Finlin et al., 2000), whose expression and function are regulated by neuronal activity in response to calcium influx through voltage-gated calcium channels (Ghiretti et al., 2014). Further, gene knockdown approaches in cultured cortical neurons and *Xenopus laevis* optic tectum established that Rem2 is a positive regulator of synapse formation and a negative regulator of dendritic complexity (Ghiretti et al., 2013; Ghiretti et al., 2014; Ghiretti and Paradis, 2011; Moore et al., 2013), suggesting Rem2 may regulate structural plasticity in an activity-dependent manner. Because Rem2 expression and signaling is sensitive to neuronal activity levels (Ghiretti et al., 2013; Ghiretti et al., 2014), and Rem2 signaling regulates changes in gene expression (Kenny et al., 2017), we hypothesized that Rem2 could be a key regulator of cortical plasticity mechanisms in the intact nervous system.

To test this hypothesis, we generated *Rem2* knockout mice to directly assess how Rem2 influences cortical plasticity in the mammalian visual system. We found that *Rem2^-/-^* mice exhibit a deficit in late-phase OD plasticity during the critical period, whereas early-phase OD plasticity and adult OD plasticity are normal. These functional deficits depend specifically on deletion of *Rem2* from excitatory neurons in the cortex. At the cellular level, we found that *Rem2^-/-^* neurons exhibit altered regulation of synaptic function and increased intrinsic excitability. Further, using sparse deletion methods, we found that the effects on intrinsic excitability are cell-autonomous, and precede the effects on synaptic regulation. Consistent with the observed increase in intrinsic excitability, we demonstrated that Rem2 also induces changes in spontaneous firing rates both in vitro and in vivo. Taken together, thesedatasuggestthatRem2 providesanewunderstanding ofthemolecular processesthatcoordinate changes inintrinsicexcitability, spontaneous firing rates, synapse regulation, and ultimately circuit function.

## Results

### Rem2 is expressed in cortex during the critical period

**Figure 1.**
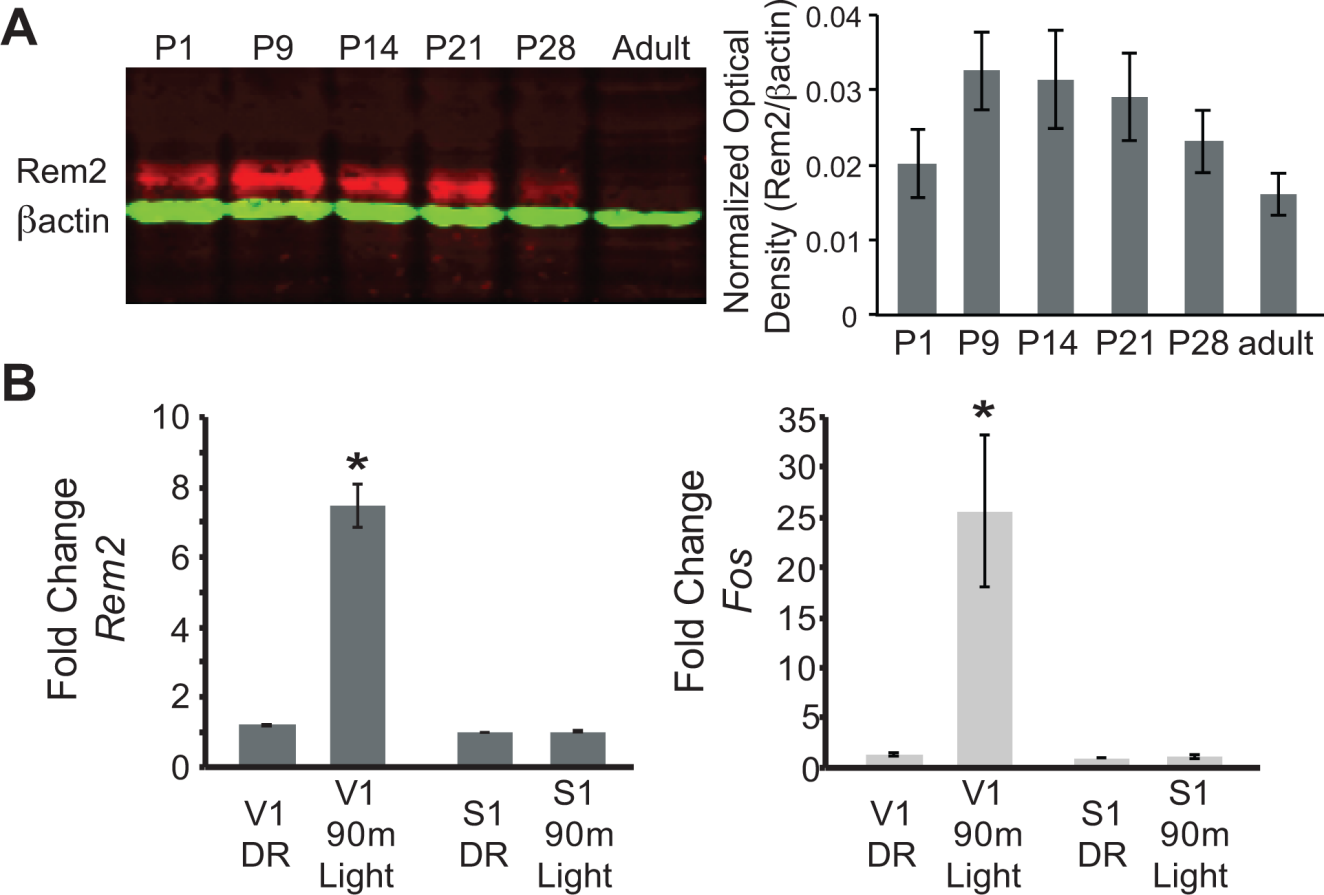
Rem2 expression is developmentally regulated and activity-dependent. (**A**) (left) Western Blot of cortical lysate from rat brains at different developmental ages detected using anti-Rem2 (1:500); anti-βactin (1:5000) was used as a loading control. (right) Quantification of relative Rem2 intensity at different developmental ages normalized to anti-βactin. Molecular Weight of REM2: ~50 kDa. Molecular weight of ACTB: 42 kDA. (**B**) Fold change in *Rem2* (left) or *Fos* mRNA expression (right) in isolated primary visual cortex (V1) or somatosensory cortex (S1) from P28 mice raised in the dark (DR, from P9-P28) or raised in the dark (P9–P28) and exposed to light for 90 m (90 m Light). N = 3 biological replicates of 4 mice in each experiment. mRNA levels were normalized to *Actb* levels and then to S1 DR condition and presented as mean ± SEM. *p<0.05 from V1 DR by one-way ANOVA followed by a Dunnett’s post hoc test.

We first sought to determine when Rem2 is expressed in the developing rodent cortex based on the hypothesis that if Rem2 is an important molecule in activity-dependent plasticity pathways, then its expression should be modulated by neural activity evoked by natural sensory stimulation. Toward this end, rat cortical lysates were harvested at different developmental time points (from postnatal day (P)1 to adult). Samples were analyzed by immunoblotting using an antibody that specifically recognizes Rem2 (Figure 1A). We found that Rem2 expression peaks in the cortex around the time of eye opening (P9-P14, eyes open at P13 in rodents), and declines near the end of the critical period (P35, (Gordon and Stryker, 1996). This result suggests that Rem2 is expressed during the developmental window in which robust synapse formation and activity-dependent refinement of cortical circuits occurs.

Given that Rem2 is expressed around eye opening (Figure 1A) and that *Rem2* mRNA is modulated by neuronal activity (Ghiretti et al., 2014) we next asked if *Rem2* mRNA expression is regulated in mammalian visual cortex by sensory experience. To address this question, mice were dark reared (DR) from P9 (prior to eye opening at P13) to P28 (during the peak of the critical period). At P28, one group of mice was exposed to 90 min of light stimulation while a separate group was kept in the dark. Following visual stimulation, primary visual cortex (V1) and primary somatosensory cortex (S1), used as a negative control, were micro-dissected and cDNA was prepared from total RNA for use in RT-qPCR experiments (Figure 1B). Compared to DR littermates, 90 min of light stimulation led to a significant and rapid increase in *Rem2* mRNA expression in V1 with no change in *Rem2* mRNA expression in S1 (Figure 1B, left). As expected, we also observed an increase in mRNA levels of the immediate early gene *Fos* in response to changes in visual experience (Figure 1B, right). These data illustrate that *Rem2* mRNA expression in V1 is modulated by visual stimulation coincident with experience-dependent development of visual cortical circuits. Interestingly, although our results demonstrate a reduction in baseline Rem2 protein expression around P28 and in adulthood (Figure 1A, compared to P9 and P14), *Rem2* mRNA expression can be modulated by visual stimulation both at P28 (Figure 1B) and in the adult visual cortex (Mardinly et al., 2016). Thus, while baseline Rem2 protein levels may decline with age, *Rem2* mRNA expression continues to be regulated by neuronal activity in discrete cell types into adulthood. These activity-dependent changes in Rem2 expression suggest that Rem2 function may be relevant throughout the life of the animal.

### Generation and validation of the *Rem2* null and conditional knockout mouse

**Figure 2.**
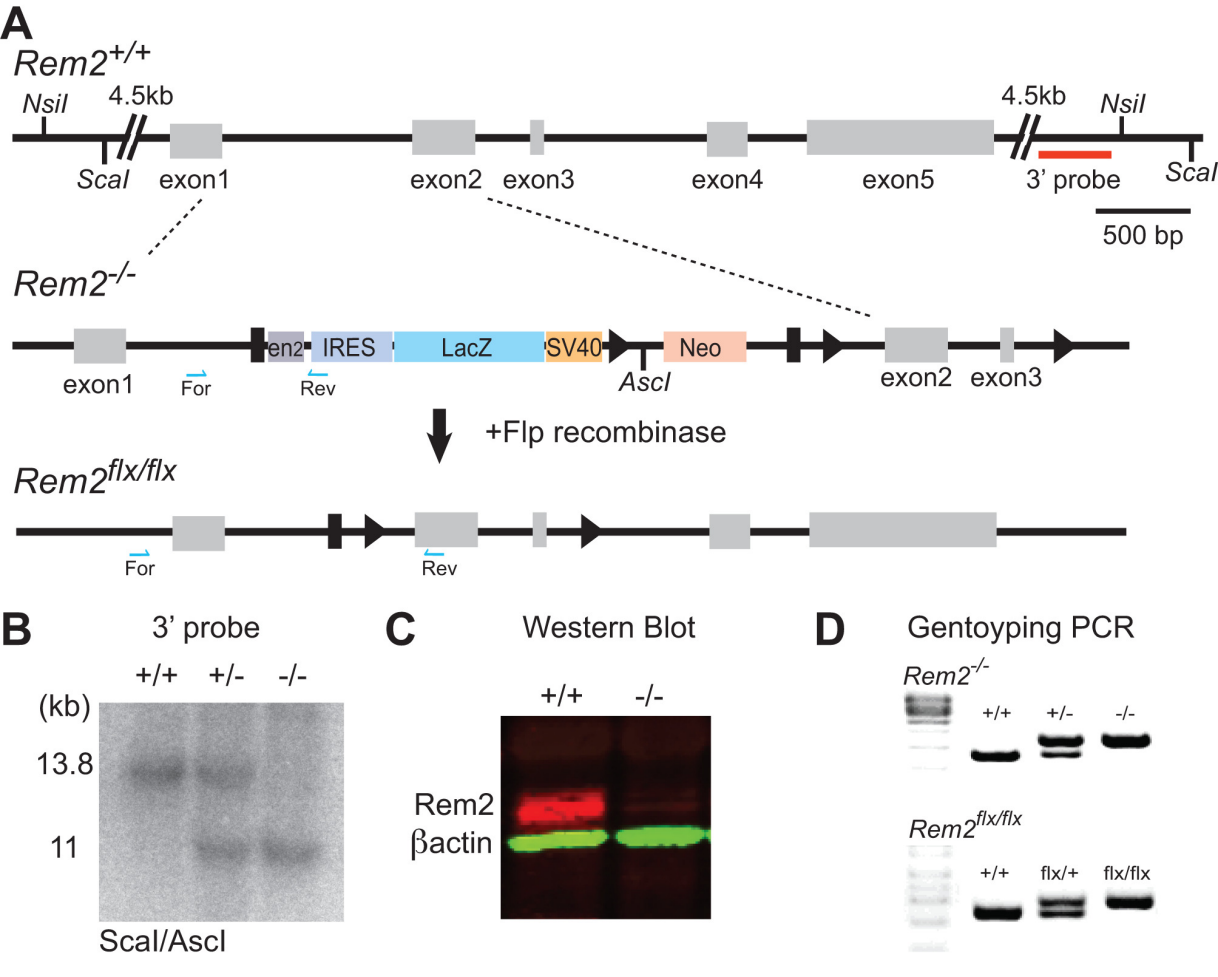
Generation of a Rem2 knockout mouse. (**A**) The *Rem2* locus (top, *Rem2^+/+^*) with exons 1–5 depicted as gray boxes. Homologous recombination was used to insert a 7.5 kb cassette containing a mouse *En2* splice acceptor sequences(EN2), IRES, a LacZ gene, a SV40 polyadenylation, and a Neo gene flanked by FRT sites (black rectangles) and LoxP sites flanking exons 2 and 3 (indicated by black triangles). Insertion of this cassette yielded a *Rem2* null allele (*Rem2^-/-^*). Following germline transmission of the insert allele, the mice were crossed to a mouse expressing Flp recombinase in the germline, which produced a floxed *Rem2* allele (termed *Rem2^flx/flx^*, bottom). Blue half arrows indicate PCR forward and reverse primer locations. Scale bar, 500 bp. (**B**) Southern blot analysis of ScaI/AscI-digested genomic DNA from mice that are *Rem2* wildtype (+/+), heterozygous (+/-) or homozygous (-/-) using the 3’ probe (red line, top A). Fragments of the predicted size (11 kb) indicate correct targeting. (**C**) Western blot analysis of *Rem2^+/+^* and *Rem2^-/-^* mice confirming the loss of Rem2 expression. Molecular Weight of REM2: 37 kDa. Molecular weight of ACTB: 42 kDA. **D**) Genotyping PCR products of genomic DNA isolated from tails of mice that were (top) wildtype (*Rem2^+/+^*), heterozygous (*Rem2^+/-^*) or homozygous (*Rem2^-/-^*) or (bottom) wildtype (*Rem2^+/+^*), heterozygous (*Rem2^flx/+^*) or homozygous (*Rem2^flx/flx^*). See Materials and methods for genotyping details.

To determine if Rem2 functions to regulate visual system plasticity in vivo, we generated a *Rem2* knockout mouse. Embryonic stem cell lines harboring a cassette with conditional potential (Figure 2A, cassette strategy originally developed by (Skarnes et al., 2011) at the *Rem2* locus were acquired from EUCOMM (Ref ID: 92501) and injected into blastocysts from which we obtained chimeras and germline transmission of this *Rem2* allele (referred to as *Rem2^-/-^*; Figure 2A middle). We verified correct insertion of the cassette at the *Rem2* locus both by Southern blot analysis (Figure 2B) and by extensive PCR followed by sequencing (Figure 2D, top). We also demonstrated that, as expected (Skarnes et al., 2011), insertion of the cassette disrupts Rem2 expression by immunoblotting (Figure 2C), confirming a null allele at the protein level. The *Rem2^-/-^* mice were also crossed with mice expressing Flp recombinase in the germline (JAX 009086), which produced a ‘floxed’ (i.e. flanked by loxP sites) *Rem2* allele (referred to as *Rem2^flx/flx^*; Figure 2A, bottom). The location of the loxP sites was confirmed by extensive PCR followed by sequencing across the entire targeted *Rem2* locus (Figure 2D, bottom).

**Figure 2—figure supplement 1.**
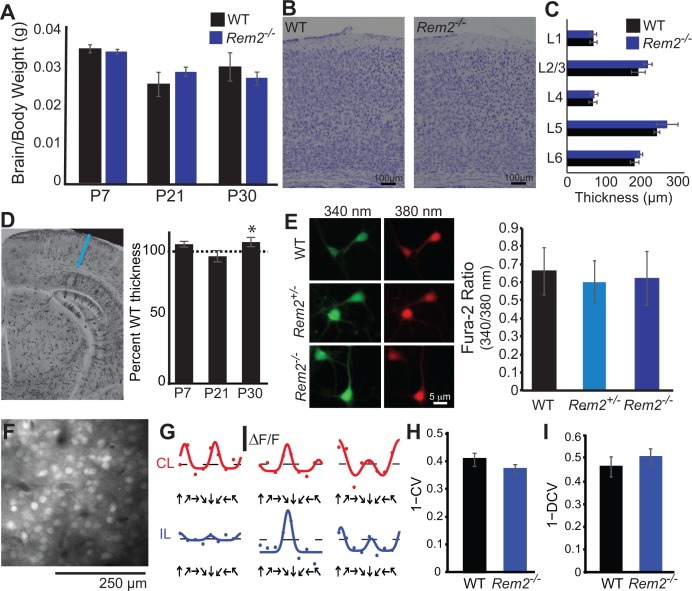
*Rem2^-/-^* mice display no gross anatomical or function cortical abnormalities. (**A**) Brain/body weight ratio of P7, P21 and P30 WT and *Rem2^-/-^* mice. (**B**) Representative images of WT and *Rem2^-/-^* Nissl stained brain slices from P16 mice (30 μm sections, scale bar 100 μm). (**C**) Cortical layer thickness measured in P16 WT and *Rem2^-/-^* mice (N = 3 animals per condition). (**D**) (Left) Representative image of visual cortex of P21 mouse brain stained with Golgi-Cox labeling. Blue line shows the distance of cortical thickness measured from the deep extent of L6 to the pial surface. (Right) Percent cortical thickness measured in P7 (N = 2 animals per condition), P21 (N = 3 animals per condition), and P30 mice (N = 4 animals per condition). Data is presented as percent thickness of *Rem2^-/-^* to WT cortical thickness. *p<0.05 by student’s *t*-test. (**E**) (Left) Representative images of Fura-2 calcium acquired at 340 nm (green) and 380 nm (red) in WT, *Rem2^+/-^*, or *Rem2^-/-^* cultured neurons. (Right) The ratio of Fura-2 signal (340 nm/380 nm) measured in WT, *Rem2^+/-^*, or *Rem2^-/-^* cultured cortical neurons. (**F**) Representative imaging field of neurons in mouse binocular visual cortex loaded with the calcium indicator dye Oregon Green BAPTA-1AM. Scale bar, 250 μm. (**G**) Example responses of 3 cells to visual stimulation (represented as ΔF/F, black vertical bar) of the contralateral (**C**) eye (top; red lines) and ipsilateral (**I**) eye (bottom; blue lines) with drifting gratings moving in different directions (black arrows). (**H**) Orientation selectivity as assessed by circular variance (1-CV) for WT (n = 59 cells, N = 7 animals) and *Rem2^-/-^* mice (n = 82 cells; N = 5 animals). (**I**) Direction selectivity as assessed by circular variance in direction space (1-DCV) for WT and *Rem2^-/-^* mice. All data is presented as mean ± SEM.

**Figure 2—figure supplement 2.**
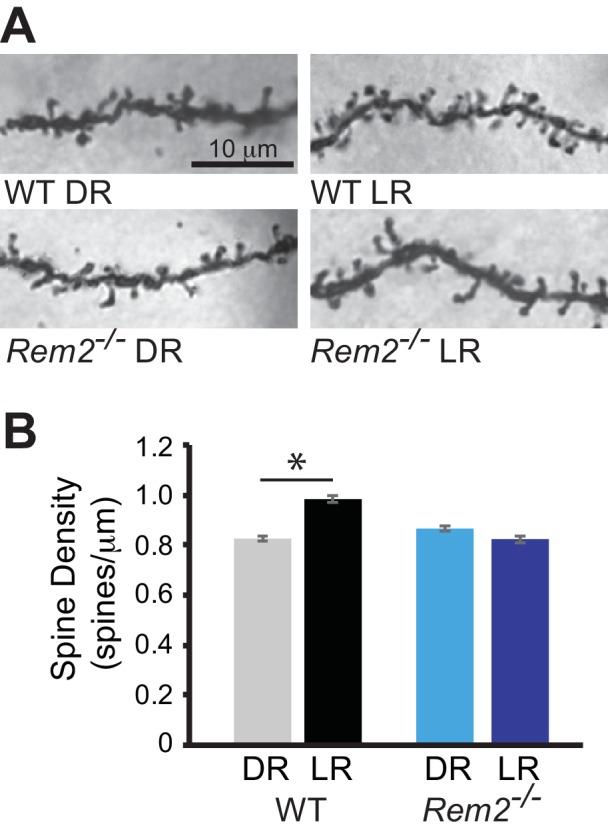
*Rem2^-/-^* mice exhibit decreased spine density in vivo. (**A**) Representative images of Golgi-cox labeled dendritic spines. Images were taken from terminal branches off the apical tree 50–100 μm from the cell soma of layer 2/3 pyramidal neurons located in primary visual cortex. Scale bar, 10 μm. (**B**) Spine density measured in wildtype and *Rem2^-/-^* mice dark reared from P9-P30 (WT DR, n = 36 neurons; *Rem2^-/-^* DR n = 36 neurons) or under normal light rearing conditions (WT LR, n = 29 neurons; *Rem2^-/-^* LR, n = 29 neurons). N = 4 animals for WT LR and *Rem2^-/-^* LR and N = 5 animals for WT DR and *Rem2^-/-^* DR experiments. Data is presented as mean ± SEM. *p<0.05 compared to WT LR by two-way ANOVA followed by Tukey post hoc.

*Rem2^-/-^* mice are viable and fertile and do not display any overt phenotypes. The genotypes of pups obtained from a *Rem2^+/-^* cross are recovered in the expected Mendelian ratio. We closely examined the brains of these animals at different developmental ages (P7, P16, P21, and P30) to determine if loss of Rem2 resulted in any overt changes in brain or cortical structure. We found no difference in the brain/body weight ratio between *Rem2^-/-^* and *Rem2^+/+^* (i.e. wildtype) littermates at P7, P21 or P30 (Figure 2—figure supplement 1A). In addition, we investigated the cortical thickness of the visual cortex at the aforementioned developmental ages. Visual cortex was identified using anatomical landmarks and measured from the deepest extent of layer six to the pial surface (just above the corpus callosum, Figure 2—figure supplement 1D, blue line). While we found no significant change in cortical thickness at P7 and P21, we did observe a small increase in cortical thickness at P30 (Figure 2—figure supplement 1D, p=0.05). We also examined cortical layer thickness by Nissl staining 30 μm sections through the visual cortex at P16 and found that layer thickness was indistinguishable between *Rem2^-/-^* and wildtype mice (Figure 2—figure supplement 1B,C). Thus, we conclude that cortical development at the gross anatomical level proceeds relatively normally despite the absence of Rem2.

A number of studies demonstrated that overexpression of Rem2 and other RGK proteins inhibit voltage-gated calcium channel function in a variety of cell types (Chen et al., 2005; Correll et al., 2008; Moore et al., 2013). Therefore, we sought to determine if resting calcium levels were perturbed by loss of *Rem2*. We prepared neuronal cultures from dissociated cortices obtained from E18 wildtype, *Rem2^+/-^*, and *Rem2^-/-^* mice. At 5 days in vitro (DIV5), neurons were loaded with the ratiometric Ca^2+^ indicator Fura-2 and images of unstimulated cells were obtained using an epifluorescence microscope (Figure 2—figure supplement 1E). Interestingly, we found no difference in the resting Ca^2+^ levels of cortical neurons obtained from wildtype, *Rem2^+/-^*, or *Rem2^-/-^* mice (Figure 2—figure supplement 1E). These data suggest that either endogenous Rem2 does not alter the Ca^2+^ permeability at rest in young cortical neurons, or that in the absence of Rem2 expression, compensatory mechanisms exist to maintain normal resting calcium levels.

To further understand the implications of *Rem2* deletion in visual system function, we examined visual response tuning properties in wildtype and *Rem2^-/-^* mice using two-photon calcium imaging (Figure 2—figure supplement 1F–I). The calcium indicator Oregon Green BAPTA-AM was bulk-loaded into cells of wildtype or *Rem^-/-^* mice (P31/32) 150 µm below the surface of the exposed binocular region of primary visual cortex (V1b; Figure 2—figure supplement 1F). Visual stimuli consisting of a series of gratings moving in different directions (0° to 360° at 45° steps) were delivered at random to each eye (Figure 2—figure supplement 1G, black arrows). No differences in measures of orientation tuning (circular variance, (Ringach et al., 2002), or in measures of direction selectivity (circular variance in direction space, (Mazurek et al., 2014) were observed between wildtype and *Rem2^-/-^* neurons (Figure 2—figure supplement 1H,I), indicating that basic visual response tuning properties are normal in *Rem2^-/-^* mice. Taken together, our data suggests that, despite the expression of Rem2 during this developmental window, deletion of *Rem2* does not interfere substantially with initial brain or circuit development in the embryonic or early postnatal period of mouse development.

In addition, previous studies in our lab have established that Rem2 functions as a positive regulator of synapse formation and dendritic spine maturation (Ghiretti and Paradis, 2011; Moore et al., 2013). Therefore, we sought to determine if *Rem2* deletion affects the experience-dependent increase in excitatory synapse density that occurs during the weeks after eye opening in visual cortex. Toward this goal, wildtype and *Rem2^-/-^* mice were either dark-reared from P9 (before eye opening) to P30 or raised under normal light-reared conditions from birth (LR, i.e. housed in a 12 hr light/12 hr dark cycle). At P30, the brains from both groups were harvested and Golgi histology performed; spine density of terminal apical branches of layer 2/3 pyramidal neurons was quantified. In wildtype mice, visual experience resulted in a significant increase in spine density relative to dark-reared animals, similar to previous reports (Figure 2—figure supplement 2: WT LR = 0.98 ± 0.01 spines/μm; WT DR = 0.83 ± 0.01 spines/μm, p=0.00012; (Valverde, 1971; Wallace and Bear, 2004). However, *Rem2^-/-^* mice did not exhibit an experience-dependent increase in spine density (Figure 2—figure supplement 2; *Rem2^-/-^* DR = 0.87 ± 0.01 spines/μm, p=0.58 compared to *Rem2^-/-^* LR). Consistent with our previous in vitro experiments, these results suggest that Rem2 functions to promote experience-dependent synapse formation in vivo.

### Rem2 is required for normal critical period OD plasticity

**Figure 3.**
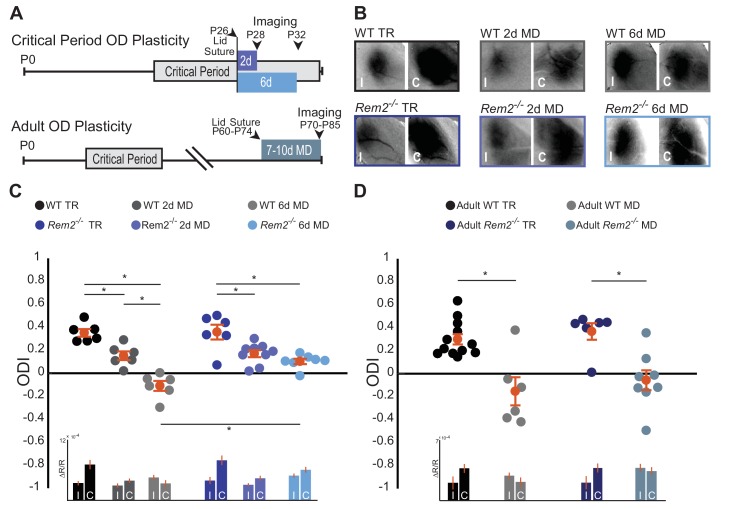
Rem2 is required for late-phase critical period ocular dominance plasticity. (**A**) Representative experimental timeline for (top) critical period ocular dominance (OD) plasticity and (bottom) adult OD plasticity. (**B**) Example fields showing intrinsic signal imaging data from the ipsilateral (**I**) and contralateral (**C**) eyes in wildtype or *Rem2^-/-^* mice that were (left) typically reared (TR) or monocularly deprived (MD) for 2 days (middle) and 6 days (right). (**C**) Ocular dominance index (ODI) for WT TR (black, n = 6), WT 2d MD (dark gray, n = 6), WT 6d MD (light gray, n = 6), *Rem2^-/-^* TR (blue, n = 6), and *Rem2^-/-^* 2d MD (medium blue, n = 9), *Rem2^-/-^* 6d MD (light blue, n = 7). Mice are shown as circles for each animal. Orange circles with error bars represent the group averages. Inset at bottom: Changes in reflectance over baseline reflectance (ΔR/R) as measured by ISI driven ipsilateral (**I**) or contralateral (**C**) eye for wildtype and *Rem2^-/-^* mice that were either typically reared or monocularly deprived. (**D**) ODI for Adult WT TR (black, n = 12), Adult WT MD (gray, n = 6), Adult *Rem2^-/-^* TR (navy blue, n = 6), and Adult *Rem2^-/-^* MD (gray blue, n = 8). Inset: ΔR/R for Adult WT and *Rem2^-/-^* TR and MD mice. Data is presented as mean ± SEM. *p<0.05 by two-way ANOVA and Tukey post-hoc. Significance comparisons for ΔR/R in C and D insets are listed in Table 1.

Given that Rem2 expression is acutely regulated by visual experience (Figure 1B), we sought to determine if Rem2 plays a role in activity-dependent processes in the visual cortex following sensory deprivation by investigating ocular dominance (OD) plasticity. We assayed critical period OD plasticity in *Rem2^-/-^* mice or wildtype littermate controls that underwent normal visual experience (typically reared, TR: 12 hr light/12 hr dark cycle) or that experienced monocular deprivation (MD) by lid suture for 2 days to measure early-phase OD plasticity or in a separate group of animals MD for 6 days to measure late-phase OD plasticity (Figure 3A top). Using optical imaging of intrinsic signals (ISI; Cang et al., 2005; Grinvald et al., 1986; Heimel et al., 2007), we measured cortical responses in the binocular portion of the visual cortex (V1b) to stimuli presented to either the left or right eye (Figure 3B, representative images). We computed the ocular dominance index (ODI) of each animal by taking the response to stimulation of the contralateral (C) eye minus the response to stimulation of the ipsilateral (I) eye, normalized to the sum of these responses [ODI = (C –I)/(C + I)].

Wildtype typically-reared littermate controls exhibited normal OD plasticity (Figure 3C). Closure of the contralateral eye for 2 days (P26-28) produced an initial, significant shift in the ODI (Figure 3C, WT TR = 0.35 ± 0.03 ODI; WT 2d MD = 0.15 ± 0.04 ODI, p=0.006). In a separate experimental group, prolonged MD for 6 days (P26-32) resulted in a further robust shift in OD toward the ipsilateral eye (Figure 3C, WT 6d MD = −0.11 ± 0.04 ODI, p=0.000001 compared to WT TR) as has been previously reported (Gordon and Stryker, 1996; Heimel et al., 2007; Mrsic-Flogel et al., 2007). Consistent with prior reports (Frenkel and Bear, 2004; Gordon and Stryker, 1996; Heimel et al., 2007; Kaneko et al., 2008b; Mrsic-Flogel et al., 2007), responses to the contralateral (deprived) eye were decreased during the early-phase of deprivation (2d MD), while responses to the ipsilateral eye increased slightly during the late-phase of deprivation (6d MD; Figure 3C, inset, Table 1).

**Table 1.** Statistical comparisons of Individual Eye Responses as measured for ocular dominance index. Statistical comparisons of changes in reflectance over baseline reflectance (ΔR/R) for ipsilateral (I) or contralateral (C) eye response vales for wildtype and *Rem2* deletion mice as measured by intrinsic signal imaging. These values correspond to the ΔR/R values displayed in Figure 3C–D and Figure 4 insets.

Statistical comparison	Ipsi p-Value	Contra p-value
WT TR vs. WT 2d MD	0.7689	0.0149*
WT TR vs. WT 6d MD	0.2242	0.0050*
WT 2d MD vs. WT 6d MD	0.0678	0.8486
*Rem2^-/-^* TR vs. *Rem2^-/-^* 2d MD	0.4259	0.0031179*
*Rem2^-/-^* TR vs. *Rem2^-/-^* 6d MD	0.3594	0.1637101
*Rem2^-/-^* 2d MD vs. *Rem2^-/-^* 6d MD	0.0247*	0.1673178
WT TR vs. *Rem2^-/-^* TR	0.5419	0.5363
WT 2d MD vs. *Rem2^-/-^* 2d MD	0.7797	0.5264
WT 6d MD vs. *Rem2^-/-^* 6d MD	0.5847	0.0096*
Adult WT TR vs. Adult WT 10d MD	0.0632	0.0257*
Adult *Rem2^-/-^* TR vs. Adult *Rem2^-/-^* 10d MD	0.0598	0.6656
Adult WT TR vs. *Rem2^-/-^* TR	0.9587	0.7801
Adult WT 10d MD vs. *Rem2^-/-^* 10d MD	0.1709	0.0998
*Rem2^+/+^*;*Emx1^Cre^* TR vs. *Rem2^+/+^*;*Emx1^Cre^* 6d MD	0.7784	0.0440*
*Rem2^flx/flx^*;*Emx1^Cre^* TR vs. *Rem2^flx/flx^*;*Emx1^Cre^* 6d MD	0.9992	0.3774
*Rem2^+/+^*;*Emx1^Cre^* TR vs. *Rem2^flx/flx^*;*Emx1^Cre^* TR	0.9990	0.9990
*Rem2^+/+^*;*Emx1^Cre^* 6d MD vs. *Rem2^flx/flx^*;*Emx1^Cre^* 6d MD	0.7446	0.5876
*Rem2^+/+^*;*Pvalb^Cre^* TR vs. *Rem2^+/+^*;*Pvalb^Cre^* 6d MD	0.3275	0.2828
*Rem2^flx/flx^*;*Pvalb^Cre^* TR vs. *Rem2^flx/flx^*;*Pvalb^Cre^* 6d MD	0.7515	0.0513
*Rem2^+/+^*;*Pvalb^Cre^* TR vs. *Rem2^flx/flx^*;*Pvalb^Cre^* TR	0.9956	0.9997
*Rem2^+/+^*;*Pvalb^Cre^* 6d MD vs. *Rem2^flx/flx^*;*Pvalb^Cre^* 6d MD	0.7564	0.7999
*Rem2^+/+^*;*Vip^Cre^* TR vs. *Rem2^+/+^*; *Vip^Cre^* 6d MD	0.0443*	0.0875
*Rem2^flx/flx^*; *Vip^Cre^* TR vs. *Rem2^flx/flx^*; *Vip^Cre^* 6d MD	0.2417	0.0554
*Rem2^+/+^*; *Vip^Cre^* TR vs. *Rem2^flx/flx^*; *Vip^Cre^* TR	0.7063	0.7400
*Rem2^+/+^*; *Vip^Cre^* 6d MD vs. *Rem2^flx/flx^*; *Vip^Cre^* 6d MD	0.5437	0.3990

*p≤0.05 by a two-way ANOVA followed by a Tukey test. All other comparisons are not significant.

*Rem2^-/-^* mice with normal visual experience (*Rem2^-/-^* TR) displayed an ODI similar to that observed in wildtype mice (Figure 3C, *Rem2^-/-^* TR = 0.35 ± 0.06 ODI, p=0.54 compared to WT TR). Closure of the contralateral eye in *Rem2^-/-^* mice for 2 days induced an initial, significant shift in the ODI similar to that observed in WT mice (Figure 3C, *Rem2^-/-^* 2d MD = 0.17 ± 0.03 ODI, p=0.008), indicating that early-phase OD plasticity is intact in the absence of Rem2. As expected, the magnitude of depression of the deprived, contralateral eye responses following 2d MD in *Rem2^-/-^* mice is similar to those observed in wildtype mice (Figure 3C, inset 2d MD, Table 1), and suggests that a weakening of synaptic strength, reminiscent of LTD, is indeed induced in either the presence or absence of Rem2. However, in contrast to early phase plasticity, late-phase OD plasticity was altered in *Rem2^-/-^* mice relative to their WT littermate controls (Figure 3C). Following 6 days of MD, *Rem2^-/-^* mice failed to display a further shift in ODI (Figure 3C, *Rem2^-/-^* 6d MD = 0.10 ± 0.02 ODI vs. WT 6d MD −0.11 ± 0.04 ODI, p=0.0008 vs. *Rem2^-/-^* 2d MD = 0.17 ± 0.03, p=0.44), indicating a deficit in late-phase OD plasticity.

A closer examination of individual eye responses revealed interesting differences between WT and *Rem2^-/-^* plasticity. Both WT and *Rem2^-/-^* mice exhibited a decrease in responses to the contralateral (deprived) eye after 2d MD (Figure 3C, inset). However, upon 6d of MD, WT mice exhibited an increase in ipsilateral (open) eye response (Figure 3C, inset WT 6d MD). This increased response has been previously attributed to homeostatic mechanisms (Kaneko et al., 2008b; Lambo and Turrigiano, 2013), which presumably interact with continued reductions in responses to the contralateral eye to promote a further shift in ODI. By contrast, *Rem2^-/-^* mice exhibited relatively equal increases in both the contralateral eye and ipsilateral eye responses with 6d MD. Thus, *Rem2^-/-^* animals exhibited a non-competitive increase in activity during late-phase MD. These results raise the possibility that Rem2 may play an important role in regulating the absolute responsiveness of the cortex to visual stimulation, such as through regulation of neuronal excitability or synaptic scaling.

In order to gain more insight into the circuit mechanisms that might underlie the observed OD plasticity deficits of *Rem2^-/-^* mice, we went on to examine adult OD plasticity. Unlike critical period OD plasticity, adult OD plasticity relies primarily on elimination of inhibitory synapses and does not require homeostatic synaptic scaling (Hofer et al., 2006; Lehmann and Löwel, 2008; Ranson et al., 2012; Sawtell et al., 2003; van Versendaal et al., 2012). To measure changes in adult OD plasticity, WT littermate controls or *Rem2^-/-^* mice underwent normal visual experience (typically reared, TR: 12 hr light/12 hr dark cycle) or monocular deprivation (MD) for 7–10 days between 10–12 weeks of age, a time well-beyond the classically defined critical period (Figure 3A bottom), followed by ISI to measure cortical responses in V1b. Wildtype mice monocularly deprived for 7–10 days in adulthood produced a robust shift in ODI from a contralateral bias to an ipsilateral bias (Figure 3D, Adult WT TR = 0.30 ± 0.04 ODI; Adult WT MD = −0.15 ± 0.12 ODI, p=0.0005). Similarly, *Rem2^-/-^* mice that underwent monocular deprivation in adulthood also produced a significant shift in ODI following 7–10 days of MD (Figure 3D, Adult *Rem2^-/-^* TR = 0.37 ± 0.07 ODI; Adult *Rem2^-/-^* MD = −0.05 ± 0.09 ODI, p=0.003). Thus, taken together these data suggest that Rem2 is required specifically for late-phase critical period plasticity.

### Rem2 is required in cortical excitatory neurons for critical period OD plasticity

**Figure 4.**
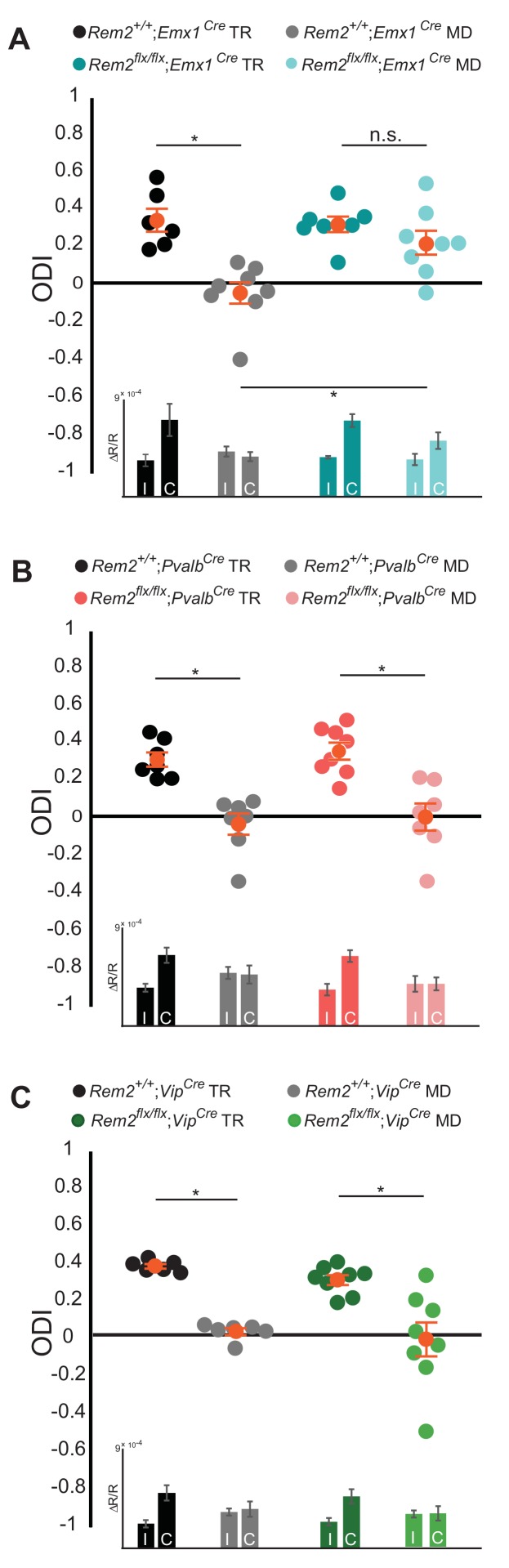
Rem2 is required in cortical excitatory neurons for ocular dominance plasticity. (**A**) Ocular dominance index (ODI) for *Rem2^+/+^; Emx1^Cre^* typically reared (TR, black, n = 6), *Rem2^+/+^; Emx1^Cre^* monocularly deprived (MD, gray, n = 8), *Rem2^-/-^; Emx1^Cre^* TR (dark teal, n = 7) or *Rem2^-/-^; Emx1^Cre^* MD (light teal, n = 8). Inset: ΔR/R for *Rem2^+/+^; Emx1^Cre^* and *Rem2^-/-^; Emx1^Cre^* TR and MD mice. (**B**) ODI for *Rem2^+/+^; Pvalb^Cre^* TR (black, n = 7), *Rem2^+/+^; Pvalb^Cre^* MD (gray, n = 7), *Rem2^-/-^; Pvalb^Cre^* TR (magenta, n = 8) or *Rem2^-/-^; Pvalb^Cre^* MD (light pink, n = 7). Inset: ΔR/R for *Rem2^+/+^; Pvalb^Cre^* and *Rem2^-/-^; Pvalb^Cre^* TR and MD mice. (**C**) ODI for *Rem2^+/+^; Vip^Cre^* TR (black, n = 6), *Rem2^+/+^; Vip^Cre^* MD (gray, n = 6), *Rem2^-/-^; Vip^Cre^* TR (green, n = 8) or *Rem2^-/-^; Pvalb^Cre^* MD (light green, n = 8). Inset: ΔR/R for *Rem2^+/+^; Vip^Cre^* and *Rem2^-/-^; Vip^Cre^* TR and MD mice. Each animal is depicted as an individual circle. Orange circles with error bars represent the averages for each group ± SEM. Data is presented as mean ± SEM. *p<0.05 by two-way ANOVA with Tukey post-hoc. Significance comparisons for ΔR/R in C and D insets are listed in Table 1.

Ocular dominance plasticity during the critical period is dependent on the proper balance of network excitation and inhibition. To probe whether Rem2 regulates the plasticity of excitatory neurons, inhibitory neurons, or both, *Rem2^flx/flx^* animals were crossed to mice directing Cre recombinase expression under the control of cell-type specific promoter elements. To assay the contribution of Rem2 expression in cortical excitatory pyramidal neurons we used the *Emx1*-Cre line (*Emx1^Cre^*, JAX #005628), where Cre expression is turned on early in embryonic development and is largely restricted to the dorsal telencephalon (Gorski et al., 2002). *Rem2^+/+^; Emx1^Cre^* or *Rem2 ^flx/flx^; Emx1^Cre^* mice were typically reared or monocularly deprived for 5–7 days as outlined above and ODI was measured using ISI. The *Rem2^+/+^; Emx1^Cre^* mice showed a pronounced shift in their ODI following 5–7 days of MD as expected (Figure 4A, *Rem2^+/+^; Emx1^Cre^* TR = 0.33 ± 0.06 ODI; *Rem2^+/+^; Emx1^Cre^* MD = −0.06 ± 0.06 ODI, p=0.0005). However, *Rem2* deletion specifically from excitatory, cortical neurons (*Rem2^flx/flx^; Emx1^Cre^*) resulted in diminished OD plasticity following MD (Figure 4A, *Rem2 ^flx/flx^; Emx1^Cre^* TR = 0. 31 ± 0.04 ODI; *Rem2 ^flx/flx^; Emx1^Cre^* MD = 0.21 ± 0.06 ODI, p=0.61). We made two important conclusions based on this data. First, Rem2 is required in the cortex to mediate proper OD plasticity, as the *Emx1* promoter does not drive Cre expression in other upstream regions of the visual system such as thalamus and retina (Gorski et al., 2002). Second, within the cortex, Rem2 is required in excitatory glutamatergic neurons for critical period ocular dominance plasticity to occur.

It is well-established that inhibitory tone plays a critical role in opening and closing the critical period for OD plasticity (Hensch and Fagiolini, 2005; Kuhlman et al., 2013; Southwell et al., 2010; van Versendaal and Levelt, 2016). Therefore, we sought to determine whether Rem2 expression in inhibitory interneurons contributes to OD plasticity. To this end, we crossed the *Rem2^flx/flx^* mice to a mouse line expressing Cre recombinase under control of the parvalbumin (*Pvalb*) promoter (*Pvalb^Cre^*, JAX#017320) as *Pvalb*^+^ interneurons are one of the most abundant interneuron subtypes in the cerebral cortex and disinhibition from interneurons plays an important role in OD plasticity (Butt et al., 2005; Kuhlman et al., 2013; Rudy et al., 2011). As expected, we observed a significant shift in the ODI in *Rem2^+/+^; Pvalb^Cre^* mice following monocular deprivation (Figure 4B, *Rem2^+/+^; Pvalb^Cre^* TR = 0.30 ± 0.04 ODI; *Rem2^+/+^; Pvalb^Cre^* MD = −0.05 ± 0.06 ODI, p=0.001). However, in contrast to the lack of OD plasticity observed in the *Rem2^flx/flx^; Emx1^Cre^* mice, deletion of *Rem2* from *Pvalb*^+^ interneurons (*Rem2^flx/flx^; Pvalb^Cre^*) had no effect on OD plasticity (Figure 4B, *Rem2^flx/flx^; Pvalb^Cre^* TR = 0.34 ± 0.05 ODI, *Rem2^flx/flx^; Pvalb^Cre^* MD = −0.01 ± 0.07 ODI, p=0.0005). These data demonstrate that Rem2 is not required in *Pvalb*^+^ interneurons for OD plasticity.

Vasoactive intestinal peptide (*Vip*) positive interneurons regulate cortical gain control during arousal (Fu et al., 2014), are integral to a disinhibitory circuit that enhances adult OD plasticity (Fu et al., 2015), and regulate visual acuity in an experience-dependent manner (Mardinly et al., 2016). Additionally, previous work demonstrated a significant increase in *Rem2* mRNA expression in *Vip*^+^ interneurons following light exposure in dark reared mice (Mardinly et al., 2016). To assay whether Rem2 is required in *Vip*^+^ interneurons for critical period plasticity we crossed the *Rem2^flx/flx^* mice to a mouse line expressing Cre recombinase under control of the *Vip* promoter (*Vip^Cre^*, JAX#010908). We observed a significant shift in the ODI in *Rem2^+/+^; Vip^Cre^* mice following monocular deprivation (Figure 4C, *Rem2^+/+^; Vip^Cre^* TR = 0.39 ± 0.01 ODI; *Rem2^+/+^; Vip^Cre^* MD = 0.04 ± 0.02 ODI, p=0.01). A similar shift was observed in the monocularly deprived *Rem2^flx/flx^; Vip^Cre^* mice, indicating that deletion of *Rem2* from *Vip*^+^ interneurons has no effect on OD plasticity (Figure 4C, *Rem2^flx/flx^; Vip^Cre^* TR = 0.32 ± 0.03 ODI; *Rem2^flx/flx^; Vip^Cre^* MD = −0.00 ± 0.09 ODI, p=0.001). Taken together these data demonstrate that Rem2 is not required in either *Pvalb*^+^ or *Vip*^+^ interneurons for critical period OD plasticity.

Interestingly, the *Rem2^flx/flx^; Emx1^Cre^* animals exhibit a smaller shift in ocular dominance after 6d MD than the *Rem2^-/-^* animals when compared to their respective littermate controls (compare Figure 3C to Figure 4A). One possible explanation for this result is that Rem2 modulates plasticity via its expression in different cell types or brain regions other than those examined by use of these particular Cre driver lines in the current study. Previous work investigating the role of *Igf1* in *Vip*^+^ interneurons in OD plasticity demonstrated that while deletion of *Igf1* in *Vip*^+^ interneurons altered inhibitory tone it did not affect OD plasticity following monocular deprivation (Mardinly et al., 2016). Thus, another possibility is that Rem2 may also function in other cell types, such as *Vip*^+^ interneurons, to help disinhibit cortical circuits and indirectly contribute to the partial shift observed in the *Rem2^-/-^* animals that is absent in the *Rem2^flx/flx^; Emx1^Cre^* mice.

### Rem2 is necessary for synaptic strengthening and maintenance

At the circuit level, monocular deprivation causes biphasic changes in cortical excitability, which underlies the observed shift in ocular dominance. Early-phase OD plasticity (1-3d MD) results in an initial decrease in responsiveness to the closed eye largely dependent on LTD-like mechanisms (Cooke and Bear, 2014; Crozier et al., 2007; Yoon et al., 2009), while the slower gain of responsiveness to the open eye during late-phase OD plasticity (4-6d MD) is due to homeostatic mechanisms including postsynaptic scaling up of excitatory synapses and intrinsic excitability homeostasis (Kaneko et al., 2008b; Lambo and Turrigiano, 2013; Mrsic-Flogel et al., 2007; Smith et al., 2009). The presence of a late-phase ocular dominance plasticity phenotype, which resulted in enhanced visual responsiveness at 6d MD in *Rem2^-/-^* animals, led us to question whether homeostatic mechanisms might be enhanced or altered in the absence of Rem2. To address this possibility, we examined the synaptic and intrinsic properties of layer 2/3 pyramidal neurons, due to their robust characterization in OD plasticity function (Keck et al., 2013; Kuhlman et al., 2013; Lambo and Turrigiano, 2013; Mellios et al., 2011; Mrsic-Flogel et al., 2007), using ex vivo slice experiments following either 2 days or 6 days of monocular deprivation.

**Figure 5.**
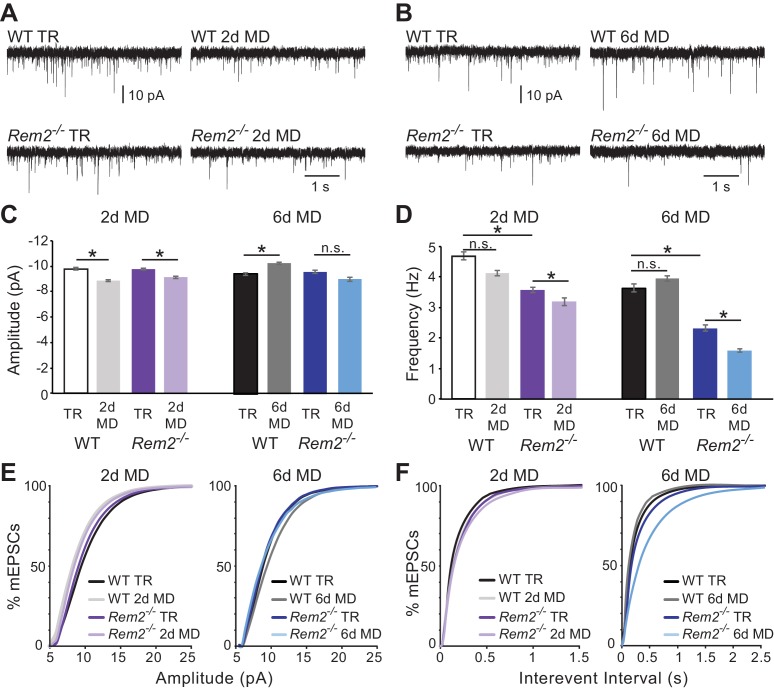
Rem2 is required for postsynaptic strengthening following 6 days of monocular deprivation. (**A**) Representative whole-cell voltage clamp recordings of mEPSCs from layer 2/3 pyramidal neurons in binocular visual cortex (V1b) of wildtype typically reared mice at P28 (WT TR), wildtype mice undergoing 2 days of monocular deprivation (WT 2d MD), *Rem2^-/-^* typically reared mice at P28 (*Rem2^-/-^* TR), or *Rem2^-/-^* mice following 2 days of monocular deprivation (*Rem2^-/-^* 2d MD). (**B**) Representative voltage-clamp traces from layer 2/3 pyramidal neurons from V1b of wildtype typically reared mice at P32 (WT TR), wildtype mice undergoing 6 days of monocular deprivation from P26-P32 (WT 6d MD), *Rem2^-/-^* typically reared mice at P32 (*Rem2^-/-^* TR) or following 6 days of MD (*Rem2^-/-^* 6d MD). (**C**) Average mEPSC amplitude recorded from wildtype or *Rem2^-/-^* layer 2/3 pyramidal neurons with normal visual experience at (left) P28 (WT TR, white, n = 25; *Rem2^-/-^* TR, dark purple, n = 29), or following 2 days of monocular deprivation (WT DR, light gray, n = 25; *Rem2^-/-^* 2d MD, light purple, n = 23) or (right) at P32 with normal visual experience (WT TR, black, n = 24; *Rem2^-/-^* TR, dark blue, n = 24) or following 6 days of monocular deprivation (WT 6d MD, gray, n = 25; *Rem2^-/-^* 6d MD, light blue, n = 25). N = 4 animals per experimental condition. (**D**) Average mEPSC frequency in wildtype and *Rem2^-/-^* mice undergoing 2 days (Left) or 6 days (Right) of monocular deprivation compared to typically reared age-matched controls. (**E**) Cumulative distribution plot of mEPSC amplitude or (**F**) Interevent Interval recorded in wildtype and *Rem2^-/-^*mice following 2 days (Left) or 6 days (Right) of monocular deprivation. Data is presented as mean ± SEM. *p<0.05, by two-way ANOVA and Tukey post-hoc for mEPSC frequency and amplitude mean data plots.

To begin, we assayed changes in postsynaptic inputs by measuring miniature excitatory postsynaptic current (mEPSC) amplitudes. Both wildtype and *Rem2^-/-^* littermates were either typically reared or monocularly deprived for 2 or 6 days starting at P26 and continuing until P28 or P32, respectively (Figure 5A,B; representative traces). Whole-cell voltage-clamp recordings were used to measure mEPSCs in layer 2/3 pyramidal neurons in acute slices. We found that 2 days of monocular deprivation in wildtype mice resulted in a significant decrease in mEPSC amplitude recorded from layer 2/3 pyramidal neurons (Figure 5A,C and E (left); WT TR = −9.8 ± 0.1 pA; WT 2d MD = −8.87 ± 0.08 pA, p=0.015) as previously reported (Lambo and Turrigiano, 2013). Similarly, recordings from layer 2/3 pyramidal neurons in cortical slices obtained from *Rem2^-/-^* mice following 2 days of MD also resulted in a significant decrease in mEPSC amplitude (Figure 5A,C and E (left), *Rem2^-/-^* P28 TR = −9.76 ± 0.08 pA; *Rem2^-/-^* 2d MD = −9.12 ± 0.08 pA, p=0.014). These data, in agreement with our 2d MD ODI data in *Rem2^-/-^* mice (Figure 3C), suggest that in the absence of Rem2, layer 2/3 cortical neurons are sensitive to the decrease in drive that occurs as a result of MD and decrease their excitatory postsynaptic strength as expected (Lambo and Turrigiano, 2013).

We next examined the consequence of 6 days of MD in the absence of Rem2 by again assaying mEPSC amplitude in layer 2/3 pyramidal neurons. We hypothesized that if *Rem2^-/-^* neurons were able to undergo homeostatic postsynaptic scaling up, we would observe an increase in mEPSC amplitude, as previously reported (Lambo and Turrigiano, 2013). As expected, we found a significant increase in mEPSC amplitude in neurons in slices obtained from wildtype animals following 6 days of MD (Figure 5B,C and E (right); WT TR = −9.46 ± 0.1 pA; WT 6d MD = −10.31 ± 0.09 pA, p=0.027). The magnitude of the effect (~10% increase following 6d MD) is consistent with previous reports (Lambo and Turrigiano, 2013). However, we failed to observe a significant increase in mEPSC amplitude in neurons from *Rem2^-/-^* visual cortex following 6 days of MD (Figure 5B,C and E (right); *Rem2^-/-^* TR = −9.64 ± 0.12 pA; *Rem2^-/-^* 6d MD = −9.04 ± 0.14 pA, p=0.535). Thus, these data demonstrate that excitatory postsynaptic scaling up is aberrant in *Rem2^-/-^* mice.

We also examined changes in mEPSC frequency in response to 2 or 6 days of MD. As expected, we found no change in mEPSC frequency in wildtype mice following either 2 days (Figure 5D,F (left); WT TR = 4.69 ± 0.13 Hz; WT 2d MD = 4.12 ± 0.1 Hz, p=0.515) or 6 days of MD (Figure 5D,F (right); WT TR = 3.65 ± 0.13 Hz; WT 6d MD = 3.95 ± 0.1 Hz, p=0.551). Interestingly, both P28 and P32 typically reared *Rem2^-/-^* mice displayed a significant decrease in mEPSC frequency when compared to their wildtype littermate controls reared under the same conditions (Figure 5D,F; *Rem2^-/-^* P28 TR = 4.13 ± 0.1 Hz, p=0.03; *Rem2^-/-^* P32 TR = −2.34 ± 0.1 Hz, p=0.0005). The frequency of mEPSC events was still further decreased following both 2 days (Figure 5E,F (left); *Rem2^-/-^* 2d MD = 3.19 ± 0.12 Hz, p=0.003) and 6 days of MD (Figure 5E,F (right); *Rem2^-/-^* 6d MD = 1.6 ± 0.05 Hz, p=0.008) in cortical neurons isolated from *Rem2^-/-^* compared to wildtype animals. These changes in mEPSC frequency could reflect any number of differences between the cortical synaptic connections in the *Rem2^-/-^* and wildtype mice in the context of sensory deprivation including changes in excitatory synapse formation, presynaptic neurotransmitter release, or synapse maintenance and pruning mechanisms.

**Figure 6.**
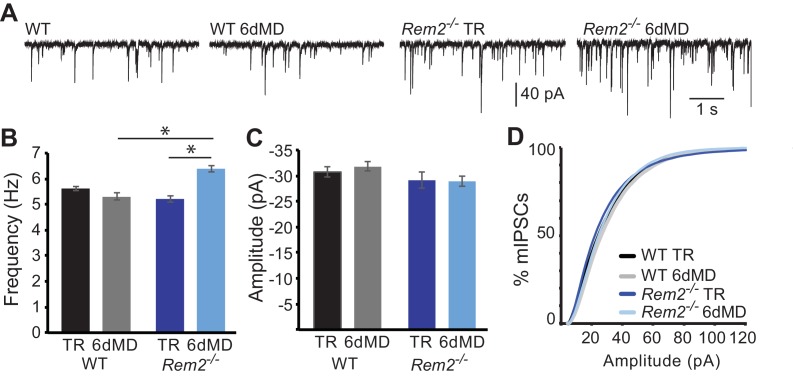
*Rem2^-/-^* does not alter inhibition in layer 2/3 pyramidal neurons. (**A**) Representative whole-cell recordings of mIPSCs from layer 2/3 pyramidal neurons in V1b in wildtype or *Rem2^-/-^* that were either typically reared (TR) until P32 or monocularly deprived (from P26-P32) for 6 days (6d MD). Quantification of average mIPSC frequency (**B**) and amplitude (**C**) in WT and *Rem2^-/-^* cells (WT TR n = 22, WT 6d MD n = 17, *Rem2^-/-^*TR n = 22, and *Rem2^-/-^* 6d MD n = 17). N = 3 animals per condition. (**D**) Cumulative distribution plot of mIPSC amplitudes in WT TR, WT 6d MD, Rem2 TR, and Rem2 MD. Data is presented as mean ±SEM. *p<0.05 by two-way ANOVA with Tukey post-hoc.

In addition to the modulation of excitatory synapses, altered neuronal activity can produce changes in inhibitory synaptic function (Hartman et al., 2006; Kilman et al., 2002; Pribiag et al., 2014). To determine if Rem2 also regulates activity-dependent changes of inhibitory synapses, we assayed miniature inhibitory postsynaptic current (mIPSC) amplitude and frequency in layer 2/3 pyramidal neurons of *Rem2^-/-^* mice (Figure 6). We found no change in mIPSC frequency (Figure 6A,B; WT TR = 5.63 ± 0.09 Hz; WT 6d MD = 5.31 ± 0.13 Hz, p=0.931) or amplitude (Figure 6C,D; WT TR = −31.04 ± 1.0 pA; WT 6d MD = −31.92 ± 0.92 pA, p=0.852) in response to 6 days of MD in wildtype neurons. Additionally, mIPSC frequency and amplitude recorded from neurons in *Rem2^-/-^* mice that were typically reared was similar to WT TR neurons, suggesting that deletion of *Rem2* does not alter baseline inhibitory synapse formation or transmission (Figure 6, (B) *Rem2^-/-^* TR frequency = 5.27 ± 0.13 Hz; (C, D) amplitude = −29.22 ± 1.47 pA, p=0.73 compared to WT TR). We did, however, observe a significant increase in mIPSC frequency, but not amplitude, in *Rem2^-/-^* mice with 6 days of MD (Figure 6B–D, *Rem2^-/-^* 6d MD frequency = 6.32 ± 0.13 Hz, p=0.03; *Rem2^-/-^* 6d MD amplitude = −29.02 ± 1.0 pA, p=0.35 compared to *Rem2^-/-^* TR). Interestingly, the increase in mIPSC frequency with 6d MD observed in layer 2/3 neurons obtained from *Rem2^-/-^* mice is concurrent with a decrease in mEPSC frequency at the same experimental time point (Figure 5D), suggesting that a change in network excitability has occurred. We explore this possibility further below by investigating spontaneous activity in cortex in *Rem2^-/-^* mice.

### Rem2 functions cell-autonomously to regulate intrinsic excitability

While we observed deficits in synaptic scaling and decreased mEPSC frequency (Figure 5) in layer 2/3 pyramidal neurons from *Rem2^-/-^* mice, these effects seemed contrary to the enhanced responsiveness observed in *Rem2^-/-^* animals by ISI following 6d MD. Specifically, the lack of synaptic scaling up would suggest that the cortex should be less responsive to visual stimulation as opposed to more responsive as observed with ISI (Figure 3C, inset). We therefore turned our focus to intrinsic excitability, which is also homeostatically regulated in layer 2/3 pyramidal neurons in response to MD, presumably in order to counteract a perturbation in network drive (Desai et al., 1999; Lambo and Turrigiano, 2013; Maffei and Turrigiano, 2008; Marder and Goaillard, 2006; Turrigiano et al., 1998).

**Figure 7.**
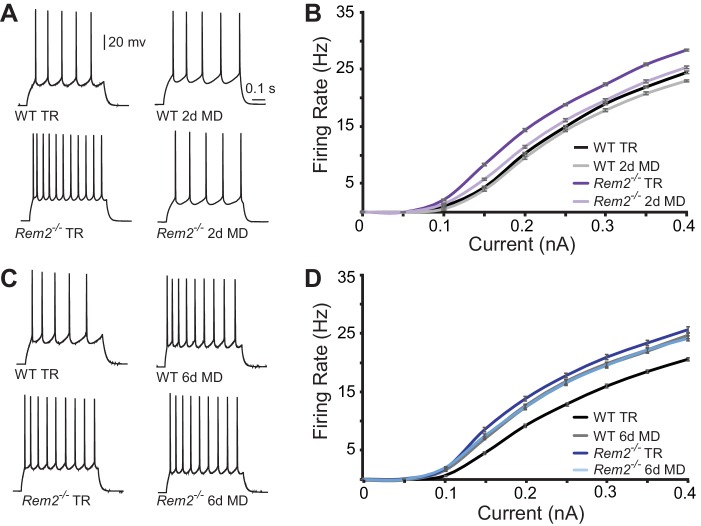
Rem2 alters the intrinsic excitability of layer 2/3 pyramidal neurons. (**A**) Example traces of evoked action potential responses (0.2 nA current injected) of neurons from WT or *Rem2^-/-^* mice either typically reared (TR) to P28 or monocularly deprived for 2 days from P26-P28 (2d MD). (**B**) Average *f-I* curves for WT TR (black line, n = 25), WT 2d MD (gray line, n = 22), *Rem2^-/-^* TR (dark purple line, n = 23) and *Rem2^-/-^* 2d MD (light purple line, n = 22) in response to normal visual experience or 2 days of monocular deprivation. N = 4 animals per experimental condition. (**C**) Examples of evoked responses from neurons of WT or *Rem2^-/-^* following at P32 that were either typically reared (WT TR and *Rem2^-/-^* TR) or monocularly deprived for 6 days from P26-P32 (WT 6d MD and *Rem2^-/-^* 6d MD). (**D**) Average *f-I* curves for WT TR (black line, n = 32), WT 6d MD (gray line, n = 22), *Rem2^-/-^* TR (dark blue line, n = 24) and *Rem2^-/-^* 6d MD (light blue line, n = 22) in response to normal visual experience or after 6 days of monocular deprivation. N = 5 mice for WT TR and 4 mice for all other conditions.

To assess changes in intrinsic excitability, we measured the frequency of action potential firing in response to a series of depolarizing current steps (*f-I* curves) applied in the presence of pharmacological blockers of synaptic transmission (Figure 7). Surprisingly, *Rem2^-/-^* mice exhibited a pronounced leftward shift of the *f-I* curve under normal rearing conditions (Figure 7), suggesting that Rem2 normally functions to inhibit the intrinsic excitability of the neuron. This shift occurred in the absence of changes in R_IN_, V_m_ or C_m_ (Table 2) between wildtype and *Rem2^-/-^* neurons (Table 2). Following 2 days of MD, wildtype neurons displayed no change in their *f-I* curve (Figure 7A–B, compare WT TR to WT 2d MD), while *Rem2^-/-^* neurons displayed decreased firing (Figure 7A–B, compare *Rem2^-/-^* TR to *Rem2^-/-^* 2d MD), such that the *f-I* curves obtained from *Rem2^-/-^* neurons were indistinguishable from wildtype (Figure 7B, compare *Rem2^-/-^* 2d MD to WT TR). In contrast, when examined after 6 days of MD, wildtype neurons shifted their *f-I* curve to the left (Figure 7C–D, compare WT TR to WT 6d MD), indicating a homeostatic increase in intrinsic excitability (Lambo and Turrigiano, 2013). However, neurons from *Rem2^-/-^* mice failed to further increase their intrinsic excitability in response to 6 days of MD (Figure 7D, compare *Rem2^-/-^* TR to *Rem2^-/-^* 6d MD). Taken together, these data suggest that Rem2 normally functions to stabilize intrinsic excitability, as removal of Rem2 causes increased intrinsic excitability (Figure 7, TR *Rem2^-/-^*). However, short and long-term deprivation (2d vs. 6d MD), in the presence or absence of Rem2, has different effects on intrinsic excitability (Figure 7). These data imply the existence of other signaling pathways, in addition to Rem2, which also function to regulate intrinsic excitability in the context of sensory deprivation and further, are responsive to the amount of deprivation that has occurred.

**Table 2.** Passive membrane properties of layer 2/3 neurons in visual cortex. Passive membrane properties including resting membrane potential (V_R_), input resistance (R_IN_), membrane capacitance (C_M_), and Tau measured in layer 2/3 pyramidal neurons in wildtype and *Rem2^-/-^* TR or MD mice for the cells assayed in Figure 7.

Experimental condition	V_R_ (mV)	R_IN_ (GΩ)	C_M_ (pF)	Tau (ms)
WT TR	−68.01 ± 0.43	95.09 ± 1.56	113.98 ± 1.70	10.78 ± 0.21
WT 2d MD	−66.78 ± 0.38	96.46 ± 2.11	108.84 ± 2.48	10.12 ± 0.15
*Rem2^-/-^* TR	−64.98 ± 0.26	111.61 ± 1.80	105.50 ± 1.95	11.44 ± 0.16
*Rem2^-/-^* 2d MD	−68.38 ± 0.38^#^	100.94 ± 2.25	109.70 ± 2.72	10.79 ± 0.27
WT TR	−64.22 ± 0.23	104.15 ± 1.64	118.55 ± 1.97	12.03 ± 0.15
WT 6d MD	−64.92 ± 0.19	109.38 ± 1.10	119.94 ± 1.46	10.77 ± 0.10^#^
*Rem2^-/-^* TR	−63.39 ± 0.43	112.06 ± 1.62	124.80 ± 2.15	13.86 ± 0.29
*Rem2^-/-^* 6d MD	−64.38 ± 0.19	99.61 ± 1.34	125.31 ± 1.61	11.96 ± 0.15
4 days post infection *Rem2^flx/flx^* + AAV-GFP	−67.48 ± 0.54	108.88 ± 3.81	101.69 ± 2.35	11.26 ± 0.35
*Rem2^flx/flx^* + AAV-GFP-CRE	−69.38 ± 0.59	101.26 ± 2.68	96.75 ± 2.59	9.66 ± 0.31
10–12 days post infection *Rem2^flx/flx^* + AAV GFP	−64.04 ± 0.56	105.87 ± 3.75	111.54 ± 3.62	11.35 ± 0.39
*Rem2^flx/flx^* + AAV-GFP-CRE	−64.21 ± 0.44	122.07 ± 3.10	102.56 ± 2.42	12.30 ± 0.37

*p≤0.05 compared to WT TR or ^#^p≤0.05 compared to *Rem2^-/-^* TR by a two-way ANOVA followed by a Tukey test. All other comparisons are not significant. For *Rem2^flx/flx^* mice, data is compared using an independent student’s *t*-test.

**Figure 8.**
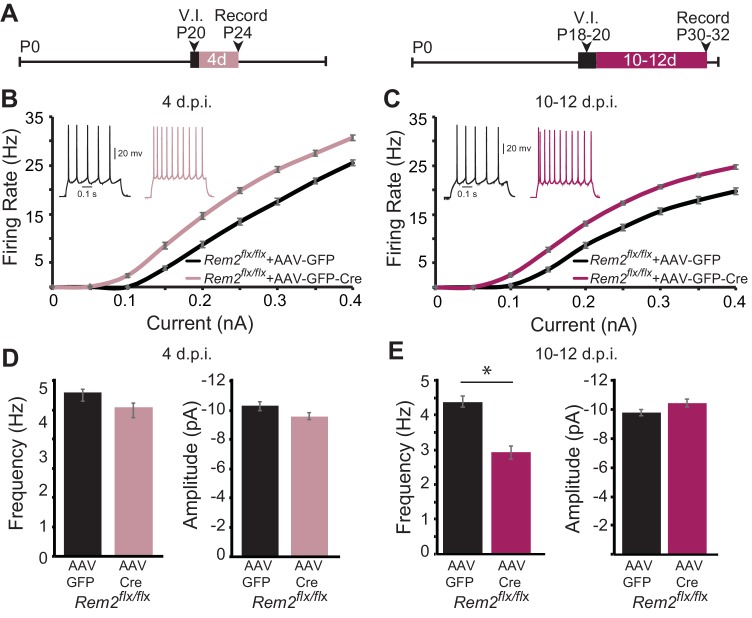
Rem2 cell-autonomously regulates intrinsic excitability in vivo. (**A**) Experimental timeline of acute, viral-mediated *Rem2* deletion in *Rem2^flx/flx^* mice. V.I., virus injection. (**B**) Average *f-I* curves recorded from neurons of *Rem2^flx/flx^* mice injected with either a control GFP (*Rem2^flx/flx^* + AAV GFP, black, n = 17) or GFP-Cre expressing virus (*Rem2^flx/flx^* + AAV GFP-Cre, mauve, n = 19) measured 4 days post infection (d.p.i.). Inset: representative traces of evoked responses measured at 0.2 nA from *Rem2^flx/flx^* + AAV GFP and *Rem2^flx/flx^* + AAV GFP-Cre neurons 4 d.p.i.. (**C**) Average *f-I* curves recorded from neurons of *Rem2^flx/flx^* + AAV GFP (black, n = 18) or *Rem2^flx/flx^* + AAV GFP-Cre (magenta, n = 20) measured 10–12 days post infection. Inset: representative traces of evoked responses measured at 0.2 nA from *Rem2^flx/flx^* + AAV GFP and *Rem2^flx/flx^* + AAV GFP-Cre neurons 10–12 d.p.i.. (**D**) Average mEPSC (left) frequency and (right) amplitude measured in *Rem2^flx/flx^* + AAV GFP (black, n = 14) or *Rem2^flx/flx^* + AAV GFP-Cre (mauve, n = 17) measured 4 days post infection. (**E**) Average mEPSC (left) frequency and (right) amplitude measured in *Rem2^flx/flx^* + AAV GFP (black, n = 22) or *Rem2^flx/flx^* + AAV GFP-Cre (magenta, n = 18) measured 10–12 days post infection. N = 3–5 animals per condition. Data is presented as mean ± SEM. *p<0.05, by two-way ANOVA and Tukey post-hoc for mEPSC frequency and amplitude mean data plots.

Given the baseline change in intrinsic excitability observed in the absence of Rem2, we next sought to address whether this shift was dependent on circuit level changes in network excitability as a result of synapse loss, or rather reflected a cell-autonomous function of Rem2. To address this question, we performed acute, sparse deletion of *Rem2* in layer 2/3 pyramidal neurons in binocular visual cortex in animals that were typically reared in a 12 hr light/12 hr dark cycle, which would allow for some level of Rem2 expression. *Rem2^flx/flx^* mice were injected at P18-20 with either a dilute control virus (AAV-GFP) or virus expressing Cre recombinase (AAV-GFP-Cre; Figure 8A). Our injection strategy causes expression of GFP (AAV-GFP), or expression of GFP and deletion of *Rem2* (AAV-GFP-Cre), from a few dozen neurons, while leaving the majority of the circuit unaffected. We previously verified using qPCR that 3–5 days of viral infection is sufficient to delete *Rem2* exons 2 and 3 (Kenny et al., 2017), Figure 1A). Acute cortical slices were then isolated either 4 days post infection (4 d.p.i., Figure 8A left) or 10–12 d.p.i. (Figure 8A right), and *f-I* curves constructed from GFP^+^, layer 2/3 pyramidal neurons to determine if *Rem2* regulates neuronal intrinsic excitability in a cell-autonomous manner.

**Figure 8—figure supplement 1.**
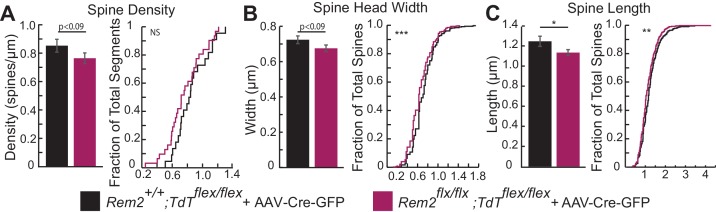
Brief loss of Rem2 results in a modest decrease in spine density and spine remodeling. (**A**) Average spine density (left) and cumulative histogram of spine densities (right) for control *Rem2^+/+^;Tdt^flex/flex^* (black, n = 22) or *Rem2^flx/^flx;Tdtflex^/flex^* (orange, n = 31) GFP-Cre epressing neurons at 11 days post injection (d.p.i). GFP-Cre expressing neurons from *Rem2^flx/flx^Tdt^flex/flex^* animals show a trend toward decreased mean spine density compared to control *Rem2^+/+^;Tdt^flex/flex^* neurons (p=0.09, two sample t-test) and a shifit in the population of spine densities toward lower values. (**B**) Average spine head width (left) and cumulative histogram (right) of spine head measurements from control *Rem2^+/+^;Tdt^flex/flex^* (black, n = 22) or *Rem2^flx/flx^;Tdt^flex/flex^* (orange, n = 31) GFP-Cre epressing neurons at 11d.p.i. GFP-Cre expressing neurons from *Rem2^flx/flx^;Tdt^flex/flex^* mice show a trend toward decreased mean spine head width compared to control *Rem2^+/+^;Tdt^flex/flex^* neurons (p=0.09, two sample t-test) and a highly significant shift in the distribution of spine head widths toward narrower spine heads (p<0.001, Kolmogorov-Smirnov test). (**C**) Average spine neck length (left) and cumulative histogram (right) of spine neck measurements from control *Rem2^+/+^;Tdt^flex/flex^* (black, n = 22) or *Rem2^flx/flx^;Tdt^flex/flex^* mice (orange, n = 31) GFP-Cre epressing neurons at 11d.p.i. GFP-Cre expressing neurons from *Rem2^flx/flx^;Tdt^flex/flex^* mice show decreased mean spine neck length compared to control *Rem2^+/+^;Tdt^flex/flex^* (p<0.05, 2 sample t-test) and a highly significant shift in the distribution of spine head widths toward narrower spine heads (p<0.01, Kolmogorov-Smirnov test). N = 5 animals per condition. Data is presented as mean ± SEM (left) and as cumulative distribution plots (right).

Surprisingly, we found that acute, sparse deletion of *Rem2* from layer 2/3 pyramidal neurons 4 d.p.i. led to a significant increase in intrinsic excitability in response to current injection (Figure 8B, *Rem2^flx/flx^* + AAV GFP-Cre 4 d.p.i.) compared to those with normal Rem2 expression (Figure 8B, *Rem2^flx/flx^* + AAV GFP 4 d.p.i.). Similarly, deletion of *Rem2* for 10–12 d.p.i. also resulted in a significant increase in intrinsic excitability (Figure 8C, *Rem2^flx/flx^* + AAV GFP-Cre 10–12 d.p.i.) compared to their wildtype littermate controls (Figure 8C, *Rem2^flx/flx^* + AAV GFP 10–12 d.p.i.). These results indicate that a primary function of Rem2 is to regulate intrinsic excitability in a cell-autonomous manner.

However, this experiment does not resolve the relationship between altered intrinsic excitability and synapse function observed in the *Rem2^-/-^* mice. Are these properties independent, or does one occur before the other? In order to determine whether the shift in intrinsic excitability was due to a preceding change in the number of functional synapses, we also measured mEPSC frequency and amplitude following 4 or 10–12 days of *Rem2* deletion. Interestingly, we found that acute deletion of *Rem2* measured 4 d.p.i. did not alter mEPSC frequency or amplitude (Figure 8D, (left) frequency: *Rem2^flx/flx^* + AAV GFP=5.59 ± 0.22 Hz; *Rem2^flx/flx^* + AAV GFP-Cre=5.10 ± 0.24 Hz, p=0.423; (right) amplitude: *Rem2^flx/flx^* + AAV GFP=−10.28 ± 0.31 pA; *Rem2^flx/flx^* + AAV GFP-Cre=−9.60 ± 0.24 pA, p=0.386). Conversely, by 10–12 d.p.i. a significant decrease in mEPSC frequency (Figure 8E, left; *Rem2^flx/flx^* + AAV GFP=4.38 ± 0.15 Hz; *Rem2^flx/flx^* + AAV GFP-Cre=2.92 ± 0.19 Hz, p=0.011) with no change in mEPSC amplitude was observed (Figure 8E right; *Rem2^flx/flx^* + AAV GFP=−9.76 ± 0.22 pA; *Rem2^flx/flx^* + AAV GFP-Cre=−10.42 ± 0.26 Hz, p=0.38).

Additionally, we quantified changes in spine density 10 d.p.i. by utilizing sparse injections of AAV-Cre-GFP into an Ai9 Cre reporter mouse line harboring a loxP-flanked STOP cassette preventing transcription of a CAG promoter-driven tdTomato (Ai9, JAX#007909) crossed to our conditional *Rem2^flx/flx^* line. Acute deletion of *Rem2* (10 d.p.i.), resulted in decreased spine density and spine head width, as well as a statistically significant decrease in spine neck length (Fig. S3). These results, together with our mEPSC data (Figure 8D–E), suggest that approximately 10 days following *Rem2* deletion there is a significant decrease in functional synapses, prior to spine shrinkage and eventual removal. Therefore, we conclude that Rem2 functions in a cell-autonomous manner to regulate intrinsic excitability irrespective of changes in excitatory synaptic density. Taken together, these data suggest that the dramatic decrease in mEPSC frequency observed in layer 2/3 pyramidal neurons in *Rem2^-/-^* mice (Figure 5D, right) most likely reflects a combination of both presynaptic and postsynaptic effects of *Rem2* deletion.

### Rem2 regulates spontaneous firing rate

**Figure 9.**
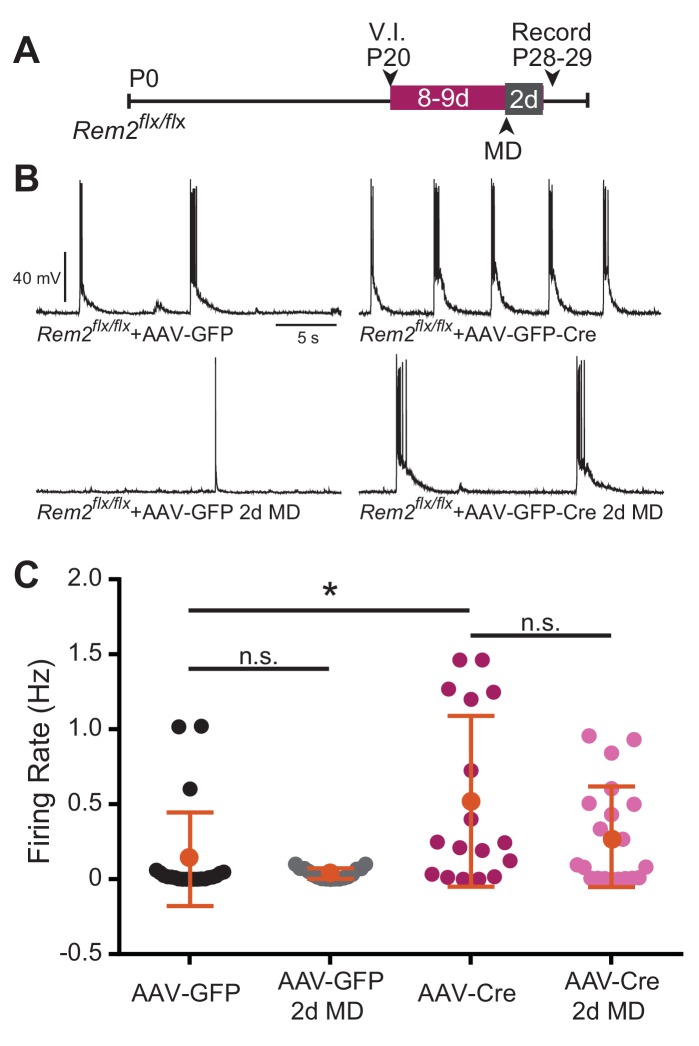
Spontaneous firing rate is regulated by Rem2 in vitro. (**A**) Experimental timeline of acute, viral-mediated *Rem2* deletion in *Rem2^flx/flx^* mice with 2 days monocular deprivation (MD). V.I., virus injection. (**B**) Examples of spontaneous firing recorded in GFP^+^ layer 2/3 pyramidal neurons in *Rem2^flx/flx^* + AAV GFP (top left, n = 22), *Rem2^flx/flx^* + AAV GFP+2 d MD (bottom left, n = 17), *Rem2^flx/flx^* + AAV GFP-Cre (top right, n = 17), and *Rem2^flx/flx^* + AAV GFP-Cre+2 d MD (bottom right, n = 20). (**C**) Average firing rate measured in layer 2/3 pyramidal neurons. Data is presented as individual data points, with the horizontal bar denoting average. N = 4 animals per condition. Error bars represent S.E.M. *p<0.05, by two-way ANOVA with Tukey post-hoc.

A neuron stabilizes firing rate through a combination of both synaptic and intrinsic homeostatic mechanisms leading to an internal firing rate set point around which the neuron operates (Turrigiano, 2011). Thus far, our data implicate Rem2 in regulating synaptic strengthening in the context of monocular deprivation (Figures 5C and 6d MD), mediating excitatory synapse function (Figure 5D, Figure 8E), and cell-autonomously regulating intrinsic excitability independent of synaptic modification (Figure 8, Figure 8—figure supplement 1). Given these data, we hypothesized that Rem2 could also regulate spontaneous firing rates in a cell autonomous manner. Thus, we recorded spontaneous activity from layer 2/3 pyramidal neurons in binocular visual cortex following acute, sparse deletion of *Rem2. Rem2^flx/flx^* mice that were reared under 12 hr light/12 hr dark housing conditions were injected at P20 with either a dilute control virus (AAV-GFP) or virus expressing Cre recombinase (AAV-GFP-Cre; Figure 9A) as outlined above. Spontaneous action potential firing was measured by whole-cell current clamp recordings 8–9 d.p.i. from acute cortical slices. We found that deletion of *Rem2* leads to a significant increase in spontaneous firing rates in layer 2/3 pyramidal neurons (*Rem2^flx/flx^* + AAV GFP-Cre=0.52 ± 0.067 Hz, p=0.008, Figure 9B top right) compared to wildtype control neurons (*Rem2^flx/flx^* + AAV GFP=0.154 ± 0.029 Hz, Figure 9B top left). Thus, Rem2 functions cell-autonomously to stabilize neuronal firing rates.

Given that we observed a significant decrease in intrinsic excitability of layer 2/3 pyramidal neurons recorded from *Rem2^-/-^* mice following 2d of MD (Figure 7B), we also sought to determine whether spontaneous activity changed significantly with acute *Rem2* deletion in the context of a similar deprivation. To address this question, a subset of *Rem2^flx/flx^* mice were subjected to 2d of MD after viral injection of either AAV-GFP or AAV-GFP-Cre (Figure 9A). In both *Rem2^flx/flx^* + AAV GFP and *Rem2^flx/flx^* + AAV GFP-Cre mice, 2d MD had no effect on spontaneous firing rates recorded from GFP + layer 2/3 pyramidal neurons (*Rem2^flx/flx^* + AAV GFP 2d MD = 0.029 ± 0.0038 Hz, p=0.8442 compared to *Rem2^flx/flx^* + AAV GFP; *Rem2^flx/flx^* + AAV GFP *Rem2^flx/flx^* + AAV GFP-Cre 2d MD = 0.284 ± 0.0038 Hz, p=0.203 compared to *Rem2^flx/flx^* + AAV GFP-Cre). These data suggest that decreased intrinsic excitability observed with 2d MD does not directly translate into changes in spontaneous firing rate in individual neurons (Figure 9C). However, these experiments were performed using slightly different genetic manipulations (*Rem2* total knockout (*Rem2^-/-^* Figure 7) versus sparse *Rem2* knockout (*Rem2^flx/flx^* + AAV GFP-Cre Figure 9). Thus, it is possible that the decrease in intrinsic excitability observed in the *Rem2^-/-^* mouse following 2d MD (Figure 7A,B) implicates additional roles for Rem2. For example, Rem2 may play a role in regulating presynaptic neurotransmitter release, which in turn influences how individual neurons response to circuit-level changes in activity.

**Figure 10.**
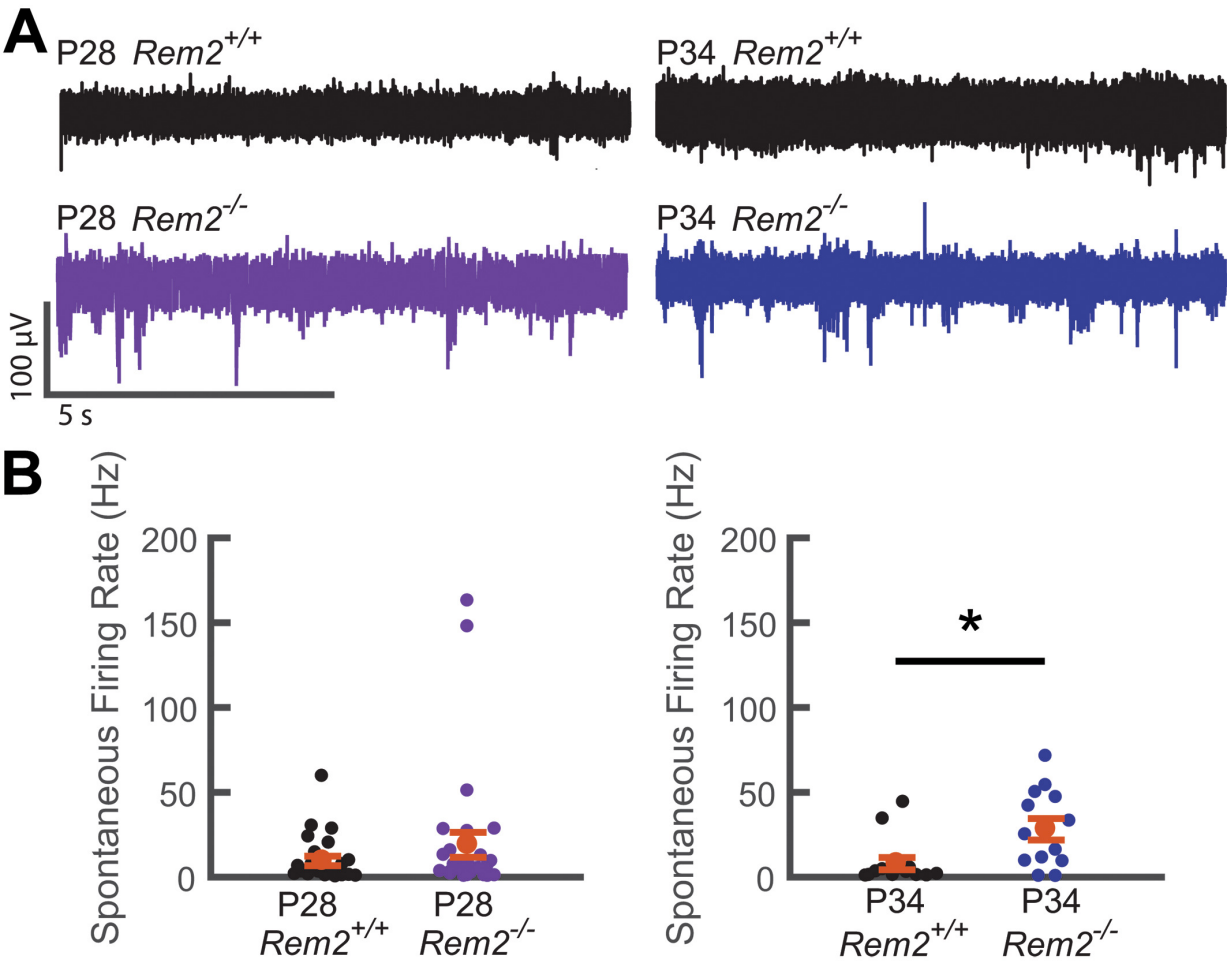
Spontaneous firing rate is regulated by Rem2 in vivo. **A**) Representative extracellular multi-unit voltage traces from L2/3 neurons in binocular visual cortex (V1b) of wildtype (top) and *Rem2^-/-^* (bottom) typically reared mice sampled during the peak (P28, left) and late (P34, right) visual critical period. (**B**) Average spontaneous multiunit firing rate of L2/3 V1b neurons sampled from anesthetized wildtype and *Rem2^-/-^* mice during the peak (Right, P28 WT, black, n = 24 sites recorded from 4 mice; P28 *Rem2^-/-^*, purple, n = 29 sites recorded from 5 mice) and late (Left, P34 WT, black, n = 14 sites sampled from 3 mice; P34 *Rem2^-/-^*, blue, n = 13 sites sampled from 3 mice) visual critical period. Each small circle represents one recording site. Orange circles and error bars are mean ± SEM. *p<0.0153 by Kruskal-Wallis test.

Given our findings ex vivo, we hypothesized that spontaneous activity could be perturbed in the intact visual cortex of *Rem2^-/-^* mice. To this end, we performed in vivo extracellular recordings of spontaneous activity from V1b of anesthetized wildtype and *Rem2^-/-^* mice during the critical period and corresponding to our recordings made in acute slices (P28 and P34; Figure 10A–B). Interestingly, we observed no significant change in spontaneous firing rate between wildtype and *Rem2^-/-^* mice at P28 (Figure 10A–B, left). However, a significant increase in spontaneous firing rate emerges at the P34 time point (Figure 10A–B, right). Thus, in the absence of Rem2, the cortical network is more active. In addition, the increase in spontaneous firing rate recorded in vivo is consistent with the cell-autonomous increase in intrinsic excitability and spontaneous firing rates observed in our ex vivo preparations. These data (Figures 7 and 9 and Figure 10) also provide a parsimonious explanation for the intrinsic signal imaging results observed in *Rem2^-/-^* animals (Figure 3C), and support the conclusion that Rem2 regulates the absolute responsiveness of the cortex to visual stimulation through regulation of intrinsic excitability.

## Discussion

The present study identifies the activity-regulated gene Rem2 as an important regulator of intrinsic excitability. Our data demonstrate that Rem2 normally functions to stabilize the inherent activity level of a neuron, as intrinsic excitability is increased following *Rem2* deletion. Consistent with this finding, we show that spontaneous firing rate is increased in both individual neurons and in the intact cortex in the absence of Rem2 (Figures 9 and 10). Based on the data presented in this study, we favor a model in which Rem2 functions to dampen intrinsic excitability during periods of robust activity. In this model, during periods of low activity, in which we expect little to no Rem2 expression, neurons alter their intrinsic excitability through other signaling pathways. As such, we propose that Rem2 acts as a stabilizer to restrict excitability in high-activity regimes in order to maintain circuit function within an appropriate physiological range.

### Rem2 regulates visual system plasticity

Our findings demonstrate that Rem2 functions in excitatory cortical neurons to mediate OD plasticity in response to long-term monocular deprivation during the critical period (Figures 3 and 4) in part through regulating intrinsic excitability and synaptic function. Consistent with this result, our electrophysiological studies demonstrate that the activity-regulated gene *Rem2* is required for homeostatic synaptic scaling up (Figure 5) and proper regulation of intrinsic excitability in vivo (Figure 7). These data, combined with the observed deficit in late-phase OD plasticity (Figure 3C) and normal adult OD plasticity (Figure 3D) support the premise that Rem2 may be required for proper homeostatic function during the critical period. Further, we suggest the intriguing possibility that Rem2 may be a key molecule required for establishing and maintaining the firing rate set point during critical period development in vivo.

While several studies have examined the contributions of synaptic scaling and ODP (Hengen et al., 2013; Kaneko et al., 2008b; Mrsic-Flogel et al., 2007; Ranson et al., 2012; Ranson et al., 2013), little is known of the effects of cell-autonomous regulation of intrinsic excitability on visual circuit function. For example, transgenic mice harboring a deletion of TNFα also display a deficit in late-phase OD plasticity (Kaneko et al., 2008b) and normal adult OD plasticity (Ranson et al., 2012). In this study, the authors demonstrate that impaired homeostatic scaling up results in a failure to increase open eye responsiveness, causing the observed deficit in OD plasticity (Kaneko et al., 2008b). However, this study did not examine a possible role for TNFα in regulation of intrinsic excitability. In contrast, *Rem2^-/-^* mice display both impaired synaptic scaling up (Figure 5) and altered intrinsic excitability (Figure 7), as well as a generalized increase of individual eye responses with 6d MD (Figure 3C). These latter results are similar to those observed following binocular deprivation, which leads to increased cortical responsiveness to both eyes (Mrsic-Flogel et al., 2007). While it may seem counterintuitive to observe impaired synaptic scaling and increased intrinsic excitability as we do in the cortex of *Rem2^-/-^* mice, it is apparently this combination of cellular phenotypes that lead to the observed increase in spontaneous firing rates observed in vivo. Thus, while it remains to be determined exactly how these two processes function synergistically to maintain proper circuit function, our discovery of the role of Rem2 in this process provides novel insight into the importance of neuronal regulation of intrinsic excitability to sculpting network output.

### Rem2 stabilizes intrinsic excitability

While a number of molecules have been implicated in regulation of synaptic scaling (Arc (Gao et al., 2010; Shepherd et al., 2006), BDNF (Kaneko et al., 2008a; Rutherford et al., 1998), Homer1a (Hu et al., 2010; Nedivi, 1999), MHCI (Goddard et al., 2007; Syken et al., 2006), and NARP (Chang et al., 2010; Gu et al., 2013), a comparable understanding of the regulation of neuronal intrinsic excitability is lacking. Rem2 is a noncanonical Ras-like GTPase, expressed throughout the cell (Ghiretti and Paradis, 2011) and is unlikely to be regulated by its nucleotide binding state (Correll et al., 2008) in contrast to canonical Ras family members. The mechanism by which Rem2 transduces signals is an open area of investigation, although it has been shown to associate with VGCC subunits (Chen et al., 2005; Finlin et al., 2006; Finlin et al., 2005; Pang et al., 2010) and CaMKII (Flynn et al., 2012; Royer et al., 2017). In addition, our previous studies demonstrated that Rem2 functions in a CaMK signaling pathway to regulate dendritic complexity (Ghiretti et al., 2014). Interestingly, CREB is a known output of CaMK signaling and has also been implicated in regulation of dendritic morphology (Redmond et al., 2002), synaptic scaling (Ibata et al., 2008; Joseph and Turrigiano, 2017), and intrinsic excitability (Dong et al., 2006; Joseph and Turrigiano, 2017). In fact, two of these studies (Dong et al., 2006; Joseph and Turrigiano, 2017) strongly suggest that neuronal intrinsic excitability is under transcriptional control.

We propose that Rem2 functions as a calcium-sensitive cytoplasmic signal transduction molecule, conveying changes at the membrane (e.g. Ca^2+^ influx) into changes in gene expression in the nucleus (e.g. CREB) to regulate intrinsic excitability. Consistent with this model, we performed RNA-sequencing to identify downstream targets of Rem2 (Kenny et al., 2017). We found that the expression of a number of ion channels, important for establishing neuronal excitability, are regulated by Rem2 signaling in an activity-dependent manner (Kenny et al., 2017). We also recently demonstrated that Rem2 is a novel inhibitor of CaMKII catalytic activity (Royer et al., 2017). Interestingly, CaMKII has been shown to modulate calcium channel function to alter intrinsic excitability (Sahu et al., 2017; van Welie et al., 2011). Thus, Rem2 signaling may regulate neuronal intrinsic excitability by controlling the composition or function of ion channels at the cell membrane.

### Rem2 in structural plasticity

Our previous findings revealed that Rem2 is also an important regulator of synapse formation and dendritic morphology in cultured neurons and *Xenopus* optic tectum (Ghiretti et al., 2013; Ghiretti et al., 2014; Ghiretti and Paradis, 2011; Moore et al., 2013). The current study also demonstrates that Rem2 regulates dendritic spine morphology in a cell-autonomous manner (Fig. S3), as well as experience-dependent changes in spine density (Figure 5D). Similar to our findings with Rem2, other activity signaling pathways also co-regulate experience-dependent circuit plasticity and synapse development (Piochon et al., 2016; Titley et al., 2017). For example, *cpg15* has been shown to be an important regulator of synapse formation and dendritic complexity and is required during the critical period for normal OD plasticity (Picard et al., 2014). Conversely, MHCI negatively regulates synapse development and deletion of MHCI or PirB, a MHCI receptor, enhances visual system plasticity in vivo (Cebrián et al., 2014; Shatz, 2009). Although the exact contribution of subtle changes in excitatory synapse density or morphology to visual circuit plasticity remains to be determined, Hofer et al., demonstrated that spine formation and turnover is dramatically affected by MD in layer five cortical neurons (Hofer et al., 2009), suggesting that an important functional correlation exists. Hence, our findings that Rem2 functions as a regulator of experience-dependent plasticity at the morphological, cellular and circuit levels provide an important step forward in connecting molecular regulators of neuronal morphology with broader circuit function.

### Conclusions

In conclusion, our in vivo analysis of Rem2 in the visual system reveals that a primary function of Rem2 signaling is to stabilize the intrinsic excitability of cortical neurons in order to maintain proper levels of network activity. In addition to cortex, Rem2 is expressed in other areas of the brain including the hippocampus, amygdala, and nucleus accumbens (Ghiretti and Paradis, 2011; Liput et al., 2016), which are critical regions for learning, memory, and addiction. Interestingly, these processes rely heavily on physiological and morphological plasticity at the level of individual neurons (El-Gaby et al., 2015; Lüscher and Malenka, 2011; Martin et al., 2000; Yager et al., 2015). Therefore, it is likely that Rem2 acts in other brain regions and throughout the life of the animal, based on transcriptional profiling experiments performed in adult animals (Liput et al., 2016; Mardinly et al., 2016). Our data indicate that Rem2 functions at the nexus of a signaling network that senses and responds to changes in neuronal activity in order to preserve proper circuit function in the face of changing sensory experience. In the future, defining these Rem2-dependent signaling mechanisms will elucidate the molecular mechanisms that instruct activity-dependent modifications of neural circuitry.

## Materials and methods

**Key resources table:** 

Reagent type (species) or resource	Designation	Source or reference	Identifiers	Additional information
Strain, strain background (*M. musculus*)	Rem2	EUCOMM	IKMC 92501	Rem2^tm1a(EUCOMM)Hmgu^
Strain, strain background (*M. musculus*)	Flp	The Jackson Laboratory	RRID:IMSR_JAX009086	B6.129S4-Gt(ROSA)26 Sor^tm1(FLP1)Dym^/RainJ
Strain, strain background (*M. musculus*)	*Emx1*Cre	The Jackson Laboratory	RRID:IMSR_JAX:005628	B6.129S2-Emx1^tm1(cre)Krj^/J
Strain, strain background (*M. musculus*)	*Pvalb*Cre	The Jackson Laboratory	RRID:IMSR_JAX:017320	B6.129P2-^Pvalbtm1(cre)Arbr^/J
Strain, strain background (*M. musculus*)	*Vip*Cre	The Jackson Laboratory	RRID: IMSR_JAX:010908	STOCK Vip^tm1(cre)Zjh^/J
Strain, strain background (*M. musculus*)	TdT Flex	The Jackson Laboratory	RRID:IMSR_JAX:007909	B6.Cg-Gt(ROSA)^26Sortm9(CAG-tdTomato)Hze^/J
Transfected construct (Adeno-associated virus)	AAV-GFP	Penn Vector Core, USA	AV-1-PV1696	AAV1.hSyn.eGFP.WPRE.bGH
Transfected construct (Adeno-associaed virus)	AAV-GFP-Cre	Penn Vector Core, USA	AV-1-PV1848	AAV1.hSyn.HI.e GFP-Cre.WPRE.SV40
Antibody	polyclonal Anti-Rem2	Santa Cruz	sc160722; RRID: AB_2179340	use 1:500
Antibody	monoclonal Anti-Bactin	Abcam	ab8226; RRID: AB_306371	use 1:5000
Software, algorithm	ImageJ software (Fiji)	NIH	RRID:SCR_003070	
Software, algorithm	ClampFit 10.2	pClamp, Molecular Devices	RRID:SCR_011323	
Software, algorithm	Matlab	Mathworks	RRID:SCR_001622	
Software, algorithm	Reconstruct	Synapse Web	RRID:SCR_002716	
Software, algorithm	Volocity 3D Image Analysis Software	Perkin Elmer	RRID:SCR_002668	

All experimental procedures involving animals were approved by the Institutional Animal Care and Use Committee at Brandeis University.

### Western blot

Rat (developmental time course, Figure 1A) or mouse (Rem2 knockout confirmation, Figure 2D) cortices were isolated and homogenized on ice in RIPA buffer +1 x Complete Protease Inhibitor Tab (Roche) using 21, 23 and 30 gage needles attached to a 3 ml syringe. Total protein concentration for each lysate was determined using a Bio-Rad protein assay and equal amounts were loaded on the gel for each condition. Lysates were mixed with homemade 3x sample buffer (6% SDS, 0.1% bromophenol blue, 150 mM Tris, pH 6.8, 30% glycerol, and 10% β-mercaptoethanol) and boiled for 7 min. The lysates were run on a 12% SDS-PAGE gel and the proteins were transferred to a nitrocellulose membrane. The membrane was probed using anti-Rem2 (1:500; Santa Cruz cat # sc160722; RRID: AB_2179340) and anti-β-actin (1:5000; Abcam cat # ab 8226; RRID: AB_306371) antibodies as a loading control. Western blots were developed using the Odyssey Infrared Imaging System (Licor).

### Visual stimulation and gene expression analysis

Young mice were reared from postnatal day 9 (P9, prior to eye opening) until P28 in a light-tight dark box (Phenome Technologies, Inc). At P28, one group was left in the dark while a second group was exposed to light for 90 min. Then mice were anesthetized with isoflurane and decapitated. Mice from the dark group were decapitated and brains removed with the use of night vision goggles. The visual and somatosensory cortices were isolated using a customized tissue punch based on age-matched anatomical reference points and RNA was extracted with Trizol Reagent (Invitrogen). DNase-free RNA was prepared and then reverse transcribed using a Random Primer Mix (New England Biolabs). Quantitative real-time PCR was performed using SYBR green detection (Clontech) on a Rotor Gene thermal cycler (Roche). Primers for *Rem2* and *Fos* were previously verified in Ghiretti et al. (2014). The PCR products were normalized to *Actb* (β-actin) and presented as fold change over baseline using the ∆∆CT method. ‘n’ represents the number of biological replicates used. Data were compiled from independent experiments each conducted in triplicate. A one-way ANOVA followed by a Dunnett’s test was used to compare experimental conditions to control or stimulation.

### Generation of Rem2^-/-^ and Rem2^flx/flx^ mice

Embryonic stem cell lines harboring a reporter-tagged insertion with conditional potential at the *Rem2* locus (referred to as *Rem2^-/-^*; Figure 2A) were acquired from the European Conditional Mouse Mutagenesis Program (EUCOMM) from the International Knockout Mouse Consortium (IKMC; Ref ID: 92501). The cassette was inserted into the first intron of the *Rem2* locus and contained a mouse *En2* splice acceptor sequence (EN2), IRES, a LacZ gene, a SV40 polyadenylation, and a Neo gene flanked by FRT sites and LoxP sites flanking exons 2 and 3 (see Figure 2A; see Skarnes et al. (2011)for details). Insertion of the cassette into the *Rem2* locus in the ES lines was verified by extensive PCR and sequencing. Embryonic stem cells were injected into C57BL/6 blastocysts using standard conditions. Injected blastocysts were surgically implanted into pseudo-pregnant foster female mice to generate chimeric offspring. Chimeras were mated to C57BL/6 females to obtain germline transmission; genotyping was performed by PCR. Correct insertion of the cassette was verified by Southern blotting (see Figure 2B). Note that for unknown reasons, β-galactosidase protein was not expressed in mice harboring the *Rem2* null allele. For routine experimentation, animals were genotyped using a PCR-based strategy. Animals harboring the *Rem2* null allele were genotyped with a forward primer in the intron spanning region of exon 1 (5’- GCTTCTTCTAGCTCCATCGTTG-3’) and a reverse primer in the inserted cassette region (5’- GGACCACCTCATCAGAAGC-3’) or the reverse primer in exon 2 (5’- AGTTGGGAAGCTATATCTTC-3’) (Figure 2A, blue arrows, D).

We observed that inbreeding of the *Rem2^-/-^* allele to the C75BL/6 strain resulted in mice with small litters and poor viability. To determine if this phenotype was due to deletion of the *Rem2* gene, we outcrossed these mice to the 129-Elite mice strain (Charles River) and found a dramatic improvement in viability and litter size. Thus, we continued to outcross the *Rem2^-/-^* allele for at least five more generations. All experiments reported in this study for the *Rem2^-/-^* allele are on this outcrossed background. We used aged-matched, littermate WT and *Rem2^-/-^* mice for all experiments. Note that in a handful of experiments for intrinsic signal imaging, a complete set of age-matched littermate controls were not always possible (see below).

To generate a *Rem2* conditional allele, the *Rem2^-/-^* mice were crossed with mice expressing Flp recombinase with a ROSA26 promoter region (RRID:IMSR_JAX:009086) resulting in exons 2 and 3 flanked by loxP sites (Figure 2A). Conditional knockout animals were genotyped for the presence of the remaining loxP site, which shifts the size of the PCR product in the *Rem2^flx/flx^* animal by 66 bp, with the forward primer (5’-. CATCCTGGCTCCAACCATGG-3’) and reverse primer (5’-CTCCGGTCCTGTCACATCAG-3’) in the intron spanning region between exons 3 and 4 (Figure 2D). These mice were also backcrossed into the 129-Elite mice for more than five generations. To generate lines with *Rem2* deleted in defined cell types, *Rem2^flx/flx^* mice were crossed to either the *Emx1*Cre line (*Emx1^Cre^*, RRID:IMSR_JAX:005628), *Pvalb-*Cre line (*Pvalb^Cre^*, RRID:IMSR_JAX:017320), or *Vip*Cre (*Vip^Cre^*, RRID: IMSR_JAX:010908). All mice were maintained as heterozygous mating pairs. Mutant mice were identified by performing PCR on tail genomic DNA (Figure 2D).

### Southern blot

Genomic DNA was isolated from the livers of mice and digested with Sca1 and Asc1 restriction enzymes. The DNA was run on a 0.8% agarose gel and transferred to a nitrocellulose membrane using a vacuum blotter (Qbiogene, TransDNA Express^TM^ Vacuum Blotter). The nitrocellulose membrane was incubated in pre-hybridization solution (50% Formamide, 5x SSPE,. 1% SDS, 5x Denhardt’s solution, 1 mg Salmon sperm DNA) at 42°C for 4 hr. The probes were labeled using α-^32^P dATP (3000 Ci/mmol; PerkinElmer Life and Analytical Sciences) and the Prime-it II Random Primer labeling kit (Stratagene) according to manufacturer’s instructions. The probes were added to hybridization buffer (50% Formamide, 5x SSPE,. 1% SDS, 1x Denhardt’s solution, 1 mg Salmon sperm DNA) and incubated overnight at 42°C. The nitrocellulose was washed three times with 2x SSC/0.1% SDS, once with 0.5x SSC/0.1% SDS and once with 0.1x SSC/0.1% SDS. The membrane was exposed overnight in a phosphorImager cassette and imaged using the STORM molecular imaging system (GE Healthcare). The inserted 7.5 kb cassette introduced an AscI site between the LacZ and Neo markers (Figure 2A). Therefore, the predicted band size with the 3’ probe, if the cassette was correctly inserted at the *Rem2* locus, is an 11 kb band for the *Rem2^-/-^* locus and a 13.8 kb band for the wildtype locus (Figure 2B) using a ScaI/AscI double digest.

### Cortical thickness and brain weight measurements

Typically reared P7, P21, and P30 WT or *Rem2^-/-^* littermates were deeply anesthetized using ketamine/xylazine cocktail (ketamine 50 mg/kg, xylazine 5 mg/kg) and perfused first with 0.1M PBS followed by 4% paraformaldehyde in 0.1M sodium phosphate buffer. Brains were carefully extracted and stored in 4% paraformaldehyde in 0.1M sodium phosphate buffer for 24 hr, then transferred to 30% sucrose for at least 24 hr. Sections were cut at 30 µm on a freezing microtome and mounted on slides coated with 2% porcine gelatin. Slides were allowed to dry at room temperature and stored at 4°C until histology was performed. Briefly, slides were washed with xylenes and then dehydrated with a series of graded ethanols (100%, 95%, and 70%). Slides were then rinsed with ddH_2_0, stained with 0.1% cresyl violet for 1 min, cleared with a grades series of ethanols, and differentiated with glacial acetic acid in 95% ethanol. Slides were then dehydrated in a series of graded ethanols, cleared with xylenes, and coverslipped using Permount (Fisher Scientific). Every second section containing visual cortex was imaged at 4X magnification using a Keyence BZX-700 microscope (Keyence). Visual cortex was identified using anatomical landmarks diagramed in the Allen Brain Atlas and cortical layers were identified using changes in cell size and density. Cortical thickness was measured from the deepest extent of layer six to the cortical surface using the ImageJ Measure function. Boundaries of cortical layers were manually drawn using ImageJ. Averages per animal are computed across all measured sections of visual cortex. Resulting measurements from *Rem2^-/-^* mice were normalized to the measurements of their WT littermate to account for differences in histology conditions. Brain to body weight ratio was calculated using the weight and brain weight measurements recorded at the time of perfusion.

### Golgi-Cox labeling

Typically reared WT and *Rem2^-/-^* littermate mice were housed in a 12 hr light/12 hr dark cycle from birth to P30. Dark reared mice were placed in a light-tight box beginning at P9 until termination of the experiment (P30). At the specified age, the mice were anesthetized with ketamine/xylazine cocktail (ketamine 50 mg/kg, xylazine 5 mg/kg) and transcardially perfused with 0.9% saline in ddH_2_0. Dark-reared mice were anesthetized in the dark and then shielded from light until after perfusion using light blocking tape (Thor Labs) to cover the eyes. Immediately following perfusion, brains were weighed and submerged in Golgi-Cox solution (FD NeuroTechnologies). Throughout all steps involving Golgi-Cox, brains were protected from light. Golgi-Cox solution was changed 24 hr after initial immersion and brains continued to be stored in Golgi-Cox solution for 7 days. After 7 days, brains were transferred to Solution C (FD Neurotechnologies) for at least 2 days. Sections were cut at 150 µm using a cryostat and immediately mounted on slides coated with 2% porcine gelatin. Histology was carried out according to the protocol supplied by FD Neurotechnologies RapidGolgi Stain Kit. Briefly, slides were washed with ddH_2_O, developed using FD Neurotechnologies Solutions D and E, rinsed in ddH_2_O, dehydrated with a graded series of ethanols, and cleared using xylenes. Slides were then coverslipped using Permount (Fisher Scientific).

### Spine analysis

Tissue sections were imaged in brightfield using a Zeiss AxioObserver microscope. For spine density quantification in wildtype and *Rem2^-/-^* mice, z-stacks of images were captured using a 60X oil objective. Neurons were sampled from layer 2/3 pyramidal neurons of the visual cortex. Visual cortex was identified using anatomical landmarks diagramed in the Allen Brain Atlas and pyramidal neurons were identified by their well-described somatic and dendritic morphology. Entire terminal apical branches approximately 50–100 µm from the soma were chosen for reconstruction and spine density quantification based on absence of artifact, lack of structural damage, and completeness of staining. Image stacks include the entire branch beginning at the branch point with the apical trunk and continuing to the branch tip. For cortical thickness measurements, single images of each section throughout the anterior-to-posterior extent of visual cortex were captured using brightfield illumination at 4X magnification using a Keyence BZX-700 microscope. All analysis was performed with the experimenter blind to genotype and rearing condition. Dendritic spines were counted manually in FIJI (NIH, RRID:SCR_003070) on z-stack images using the Cell Counter plugin. A dendritic spine was identified as any protrusion from the dendritic segment at least 0.5 μm in length or greater. Dendritic segment length was measured using the NeuronJ plugin. A total of 1500 μm of dendritic segment was counted for the WT and *Rem2^-/-^* TR segments and a total of 2000 μm of dendritic segment was quantified for the WT and *Rem2^-/-^* DR images, for an average of 50 μm segment measured per neuron.

For spine measurements following acute deletion of Rem2, both *Rem2^+/+^; TdT^flex/flex^* and *Rem2^flx/flx^; TdT^flex/flex^* mice were injected with 50 nL (diluted 1:250) AAV-GFP-Cre. Ten days post infection, animals were anesthetized with ketamine/xylazine cocktail (ketamine 50 mg/kg, xylazine 5 mg/kg) and transcardially perfused with 4% paraformaldehyde. Sections were cut at 50 µm using a freezing microtome and immediately mounted on slides and covered using Fluoromout G media. Tissue sections were imaged using Nikon Eclipse Ti3 microscope and z-stacks of images were captured using a 60X oil objective. Neurons were sampled from layer 2/3 pyramidal neurons of the visual cortex as descripted above. Entire terminal apical branches approximately 50–100 µm from the soma were imaged and 50 µm segments were chosen at random for spine density quantification. Spine density, head width, and neck length from both *Rem2^+/+^; TdT^flex/flex^* + AAV GFP-Cre and *Rem2^flx/flx^; TdT^flex/flex^* + AAV GFP-Cre mice was quantified using Reconstruct (Synapse Web Reconstruct RRID:SCR_002716, Fiala, 2005).

### Calcium imaging

Dissociated cortical neurons from embryonic day 16 (E16) mice from a *Rem2^+/-^* x *Rem2^+/-^* mating were plated on 12 mm glass coverslips at a density of approximately 70,000/cm^2^ and grown in glia-conditioned Neurobasal media with B27 supplement (Invitrogen). E16 littermates were dissociated side-by-side and plated on individual coverslips and subsequently genotyped by PCR to determine whether the cultured neurons were *Rem2^+/+^, Rem2^+/-^*, or *Rem2^-/-^*. Free resting calcium measurements were conducted 5 days after plating (DIV 5). Cortical neurons were incubated with 1 µM Fura2-AM (Invitrogen) in Tyrode’s solution containing 0.1% bovine serum albumin for 30 min at 37°C. The neuronal culture was then washed with Tyrode’s solution and incubated for an additional 30 min at 37°C in the same solution for de-esterification. After the incubation period, the coverslip was washed once more with Tyrode’s solution and mounted on an imaging chamber containing the same solution. Fura-2 fluorescence images were acquired at 37°C on an Olympus IX-70 inverted microscope using a 20 × 0.7 NA objective (Olympus UPlanApo) and a cooled CCD camera (Orca R2, Hamamatsu) controlled by Volocity 3D Image Analysis software (RRID:SCR_002668,Perkin Elmer). Fluorophore excitation was achieved using a mercury lamp and spectral separation for excitation and emission was obtained using a fura-2 filter set (Brightline Fura2-C, Semrock). Fura-2 fluorescence images with excitation centered at 340 nm and 387 (26 and 11 nm bandwidth, respectively) and emission collected at 468–552 nm were acquired sequentially using a motorized filter-wheel (Prior Scientific).

### Lid suturing

Wildtype or *Rem2^-/-^* littermates were either not surgerized or monocularly lid sutured between P25-P27 for a duration of either 2 days (2d MD) or 5–7 days (6d MD). Mice were anesthetized using either a ketamine/xylazine cocktail (ketamine 50 mg/kg, xylazine 5 mg/kg) or 2.0% isoflurane delivered using a SomnoSuite vaporizer system (Kent Scientific). The surgical area was cleaned thoroughly using povidone pads (Dynarex), with care taken to avoid contact with the eye. The eye to be suture was rinsed using bacteriostatic saline and coated with a thin film of antibacterial ophthalmic ointment prior to suturing. Lid margins were trimmed and the lid was closed with 2–3 mattress sutures using silk suture thread. Additional antibacterial ointment was applied to the sutured lid. Sutures were checked daily and if not intact animals were not used.

Lid suture for adult OD plasticity experiments was completed as above, but with suturing occurring instead between the ages of P60-74.

### Intrinsic Signal Imaging

Mice aged between P31 and P33 underwent intrinsic signal imaging (ISI) for critical period OD plasticity experiments and adult mice (10–12 weeks of age) underwent ISI for adult OD plasticity experiments. Experimental protocols did not differ between juvenile and adult groups. The experimenter was blind to genotype until the conclusion of the experiment and data analysis. The genotype of each mouse was confirmed postmortem. Mice were prepared for ISI experiments in littermate cohorts such that littermate controls were used for both experimental factors (deprivation and genotype). In the event that a littermate was not usable, such as an open eyelid suture or death during an experiment, the remaining littermate was included in our data. Analysis of our results with these mice removed resulted in the same significant relationships between groups that we report in the manuscript.

Anesthesia was induced using 4% isoflurane (100 mL/min) in an anesthesia chamber delivered using a SomnoSuite (Kent Scientific) and anesthesia was maintained at 2% isoflurane during surgical procedures. Isoflurane anesthesia was supplemented with a single dose of chlorprothixene (10 mg/kg). First, mice that had previously undergone lid suture had sutures removed and the eye re-opened. Each sutured eye was closely inspected for health and clarity; any mice with clouding of the eye or infection of the surgical area were euthanized.

In preparation for ISI, an incision was made on the scalp and skin resected to expose the skull. The skull was cleared of overlying tissue and dried to allow for secure attachment of a small headpost using adhesive (ZAP Gel, Pacer Technologies). Following placement of the headpost, mice were headfixed and the skull was thinned over a wide area containing visual cortex contralateral to the deprived eye (if applicable) using a scalpel (blade 15) until transparent under saline. The location of visual cortex was estimated as 3.0 mm lateral to the midline and 1 mm anterior to the lambda suture. After thinning, an optical widow was created to stabilize the skull and provide a flat imaging surface by applying 2% agarose to the skull and pressing a 5 mm coverslip into the agarose. This window was sealed and affixed to the skull using cyanoacrylate adhesive.

Following surgical procedures, anesthesia was reduced to between 0.7% and 1.0% for imaging. Anesthesia level was kept as low as possible without leading to pain response in the mouse. Before ISI, the brain was imaged using green light (575 nm) to serve as a record of the imaging field’s clarity and health. Intrinsic signal imaging was performed under red light (675 nm) and images were captured at a rate of 30 Hz by a Dalsa 21–01 M60 camera. Mice were shown 20 repetitions of drifting grating stimuli at either 0° or 90° of 100% contrast at 2 Hz temporal frequency and 0.04 cycles/degree spatial frequency lasting for 10 s with an inter-stimulus interval of 15 s. A blank control screen of equal average luminance to the drifting gratings was also presented as a control stimulus. Stimuli were presented with stimuli to each eye alternately, with visual input being blocked to the opposing eye using opaque, flexible light-blocking material.

All image analysis was performed using custom Matlab (Mathworks) scripts written in the Van Hooser Lab accessible via GitHub (Van Hooser, 2018), https://github.com/VH-Lab/vhlab_vhtools/). Acquired images were mean filtered, blank screen subtracted, and summed across all frames. A region of interest over binocular visual cortex, the area of the cortex responding to stimulation of the ipsilateral eye, was manually drawn. An ocular dominance index (ODI) was calculated as follows: $ODI=\frac{\left(R_{C}-R_{I}\right)}{\left(R_{C}+R_{I}\right)}$where R_C_ is the response to stimulation of the eye contralateral to the imaging window and R_I_ is the response to stimulation of the eye ipsilateral to the imaging window. Individual animal responses are plotted on each graph as well as mean ± SEM. Statistical significance was calculated using a two-way ANOVA with Tukey post-hoc. Significance comparisons for ΔR/R are listed in Table 1.

### Virus injections

The AAV-GFP (AAV1.hSyn.eGFP.WPRE.bGH; AV-1-PV1696) and AAV-GFP-Cre (AAV1.hSyn.HI.eGFP-Cre.WPRE.SV40; AV-1-PV1848) constructs were obtained from the Vector Core facility at the University of Pennsylvania. Mice age P18-P20 were anesthetized with a cocktail of ketamine (100 mg/kg) and xylazine (10 mg/kg), mounted on a stereotaxic frame, and the scull exposed. Primary binocular visual cortex was targeted by using the mouse brain atlas after adjusting for the lambda-bregma distance for age. A small 1–2 mm in diameter hole was drilled in the skull and a glass micropipette delivered 50 nL AAV (diluted 1:250) 150 μm below the dural surface. The scalp was sutured and betadine was applied. Animals recovered on a heating pad and were returned to the animal facility until use.

### In vitro electrophysiology

Whole-cell patch clamp recordings were performed on layer 2/3 pyramidal neurons in binocular primary visual cortex (V1b). Mice were anesthetized with isoflurane, decapitated, and the brains removed with the head immersed in ice-cold, choline-based cutting solution. The choline cutting solution contained (mM) 25 NaHCO_3_, 1.25 NaH_2_PO_4_·H_2_O, 2.5 KCl, 7 MgCl_2_·6 H_2_O, 25 glucose, 0.5 CaCl_2_, 110 C_5_H_14_ClNO, 11.6 ascorbic acid, and 3.1 pyruvic acid. Coronal slices (300 μm) were cut on a vibrating microtome (Leica) and allowed to recover at 37°C for 29 min followed by an additional 29 min at room temperature before recording. V1b was identified using the mouse brain atlas and the anatomical landmarks of the shape and morphology of the white matter (Lambo and Turrigiano, 2013; Maffei and Turrigiano, 2008). Layer 2/3 pyramidal neurons located in the center of V1b were selected for recordings to avoid boundary regions identified with a 40x immersion lens. Recordings were obtained at 34°C with a Multiclamp 700B amplifier and a Digidata 1440A digitizer controlled by pClamp10 software. R_s_ and R_IN_ were monitored throughout experiments using Lab Bench (Clampex 10.2, RRID:SCR_011323) and cells that exhibited changes of more than 20% in either of these parameters throughout the course of the recording were discarded. For AMPA-mediated mEPSC recordings the external solution contained (in mM): 125 NaCl, 26 NaHCO_3_, 2.3 KCl, 1.26 KH_2_PO_4_, 2 CaCl_2_, 2 MgSO_4_, 10 glucose, 1 µM tetrodotoxin (TTX, Abcam Biochemicals), 50 µM DL-2-Amino-5-phosphonopentanoic acid (APV; Sigma Aldrich), and 50 µM Picrotoxin (Sigma). The internal pipette solution contained (in mM): 135 Potassium gluconate, 10 HEPES, 2 MgCl2, 3 Na_2_ATP, 0.3 Na_2_GTP, and 10 Phosphocreatine (pH 7.3, adjusted with KOH). Rhodamine was included in the recording pipette to verify neuronal morphology at the conclusion of the recording period. The membrane potential was held at −70 mV and events were filtered at 1 kHz. Data was recorded in 3 epochs at 100 s each for a total duration of 300 s per cell. mEPSCs were then evaluated offline in Clampfit 10.2 (Molecular Devices). Detection criteria of mEPSC events included amplitudes > 5 pA and rise times < 3 ms. To measure action potential firing rates (*f-I* curves), a series of step pulses (duration 600 ms) from −200 to 400 pA in 50 pA increments were delivered from rest in the presence of 50 µM APV, 25 µM DNQX, and 50 µM Picrotoxin. A small bias current was injected to maintain V_m_ at −70 mV in between depolarizations. Measures of mIPSCs were done in the presence of 1 µM TTX, 50 µM APV, and 10 µM DNQX (Sigma Aldrich) to isolate inhibitory postsynaptic currents. Patch pipettes (3–5 MΩ) were filled with intracellular solution containing (in mM) 120 CsCl, 10 HEPES, 1 EGTA, 0.1 CaCl_2_, 1.5 MgCl_2_, 4 Na_2_ATP, and 0.3 Na_2_GTP (pH 7.3, adjusted with CsOH). Spontaneous action potential firing was measured in the presence of active ACSF containing (in mM) 125 NaCl, 26 NaHCO_3_, 3.5 KCl, 0.5 MgCl_2_, 1 NaH_2_PO_4_H_2_O, 0.5 Na^+^ Ascorbate, 1 CaCl_2_, and 10 glucose. Data is presented as mean ±S.E.M. Statistical significance was calculated using a two-way ANOVA and Tukey post hoc analysis for averaged data. Kolmogorov-Smirnov test was used to test for comparison in the cumulative distribution plots.

### In vivo extracellular recordings

Wildtype and *Rem2*^-/-^ littermate mice were prepared for in vivo extracellular electrophysiological recordings at either age P28-29 or P34. Anesthesia was induced using 4% isoflurane (100 mL/min) in an anesthesia chamber delivered using a SomnoSuite (Kent Scientific) and maintained at 2% isoflurane during surgical procedures. Isoflurane anesthesia was supplemented with a single dose of chlorprothixene (10 mg/kg). Each mouse also received a single injection of dexamethasone (3 mg/kg) intraperitoneally.

In preparation for recording, an incision was made on the scalp and skin resected to expose the skull. The skull was cleared of overlying tissue and dried to allow for secure attachment of a small headpost using adhesive (ZAP Gel, Pacer Technologies). Following placement of the headpost, mice were head-fixed and an approximately 3 mm wide craniotomy was performed over visual cortex (3.0 mm lateral, 1.0 mm anterior to lamda) to expose the brain’s surface and leaving the dura intact. A second small craniotomy was drilled over the frontal cortex of the hemisphere contralateral to the recording site for placement of a cholorided silver wire used as a reference electrode, which was inserted into the brain and anchored in place using ZAPGel adhesive. Following surgical procedures, anesthesia was reduced to between 0.7% and 1.0% for recording. Anesthesia level was kept as low as possible without leading to pain response in the mouse.

A single low impedance tungsten electrode (0.1 MΩ, World Precision Instruments TM33B01) was lowered 50 µm into the brain using a micromanipulator (Sutter Instruments MP-285) and allowed to settle for at least 30 min. Receptive fields were mapped manually using circular patches of drifting gratings. Visually-driven responses to these mapping stimuli in the center-most 20 degrees of the visual field were used to define binocular visual cortex. When binocular visual cortex was located, recordings were made of visual responses to drifting grating stimuli of varying direction of motion (30-degree steps) and spatial frequency (0.02, 0.05, 0.1, 0.2, 0.5, 1.0) as well as a luminance-matched grey screen. Recordings were limited to the first 400 µm of depth to restrict data collection to layer 2/3 of the visual cortex. Each site was at least 50 µm away from the previous site in the Z dimension. All stimuli were presented on a CRT monitor (Sony GDM520) 35 cm away from the mouse’s eyes and all stimuli. All recordings were made using an Intan Technologies (Los Angeles, CA) C3100 board with RHD2000 amplifier/digitizer board and custom electrode adaptor. Stimulus on times were recorded using Spike2 (Cambridge Electronic Design, LLC) and custom scripts written by members of the Van Hooser Lab (Van Hooser, 2018), https://github.com/VH-Lab/vhlab_vhtools/). Stimuli were designed using the Psychophysics Toolbox (Brainard, 1997; Pelli, 1997) and a custom suite of Matlab tools (Van Hooser, 2018), https://github.com/VH-Lab/vhlab_vhtools/). Spontaneous activity was analyzed during all grey screen presentations. Spontaneous firing rate was calculated as the average spikes/sec across all recorded bouts of spontaneous activity. All analyses were performed using custom Matlab functions written by the Van Hooser Lab via GitHub (Van Hooser, 2018), https://github.com/VH-Lab/vhlab_vhtools/; copy archived at https://github.com/elifesciences-publications/vhlab_vhtools).

## Additional files

Transparent reporting form

### Data availability

Imaging data is available at http://www.vhlab.org/data. All other data generated or analyzed during this study is included in the manuscript and supporting files.
