# Supplementary material for: Rem2 stabilizes intrinsic excitability and spontaneous firing in visual circuits
Source: eLife. 2018 May 29;7:e33092. doi: 10.7554/eLife.33092 (PMC6010341; doi:10.7554/eLife.33092)
Supplement: Peer review history

# Decision letter

**DOI:** 10.7554/eLife.33092.020

**Affiliations:** RIKENJapan

In the interests of transparency, eLife includes the editorial decision letter and accompanying author responses. A lightly edited version of the letter sent to the authors after peer review is shown, indicating the most substantive concerns; minor comments are not usually included.

Thank you for submitting your article "Rem2 regulates distinct homeostatic mechanisms in visual circuit plasticity" for consideration by *eLife*. Your article has been favorably evaluated by Andrew King (Senior Editor) and three reviewers, one of whom, Yukiko Goda (Reviewer #1), is a member of our Board of Reviewing Editors.

The reviewers have discussed the reviews with one another and the Reviewing Editor has drafted this decision to help you prepare a revised submission.

Summary:

Moore et al. explore a function for Rem2, an activity-regulated small GTPase, in ocular dominance plasticity and its underlying cellular mechanisms. Using constitutive and conditional *Rem2* KO mice, the authors show the involvement for Rem2 in the late-phase ocular dominance (OD) plasticity where Rem2 is required in cortical pyramidal neurons but dispensable in *Pvalb^+^* or *Vip^+^* interneurons. Furthermore, Rem2 plays a novel, cell-autonomous role in regulating intrinsic excitability of neurons. Whereas the experiments are nicely designed and the data are solid, the link between the role of Rem2 in OD plasticity and the observed alterations in synaptic and intrinsic excitability upon loss of Rem2 could be strengthened. Towards this end, the following points require full consideration, one involving additional experiments and others involving careful revision of the text to provide a balanced interpretation of the results while toning down some of the claims.

Essential revisions:

1) The effect of Rem2 deletion and MD on intrinsic excitability of pyramidal neurons seems to be biphasic, showing a decrease 2 days after MD and an increase in the following days that re-normalizes the I/F function to the baseline. The authors cannot claim that the "neurons from *Rem2^-/-^* mice failed to further increase their intrinsic excitability in response to 6 days of MD", as they do increase in comparison to 2d of MD. Furthermore, an increase in intrinsic excitability was also seen after 6 days of MD in WT mice (Figure 6). The important question is why *Rem2* KO neurons show reduced activity 2d after MD, while neurons from WT mice do not. Since 2d of MD corresponds to the initial depression of visual responses in OD plasticity (Figure 3) and can also be explained by Hebbian-like long-term depression, it is not clear how this change can be stated as homeostatic. The reduction in excitability following 2d could contribute to LTD and the authors should explore this possibility experimentally. It would be important to measure spontaneous spiking activity, in addition to intrinsic excitability measurements, in order to understand the full picture.

2) The finding that Rem2 can regulate intrinsic excitability in a cell-autonomous manner and in the absence of sensory manipulation (Figure 7) seemingly contradicts the initial finding that Rem2 mRNA expression does not increase without sensory experience in dark-reared animals (Figure 1). This would seem to be an indication that Rem2 function is likely complicated, warranting further exploration of Rem2's role in critical period plasticity. Either tone down the claims to account for possible different interpretations, or if we are wrong, clarify how we are misunderstanding this.

3) *Rem2* KO mice display early-phase OD plasticity to a similar extent as WT mice but exhibit a deficit in late-phase OD plasticity. Specifically, upon 6 days of MD (Figure 3C), WT mice increase the ipsilateral eye response but not the contralateral eye response, while *Rem2* KO mice increase both ipsilateral and contralateral eye responses that result in a lack of further shift in ocular dominance index in late-phase OD plasticity. Although this suggests that Rem2 normally suppresses the increase specifically of the contralateral eye responses relative to the ipsilateral eye responses, it is not obvious how these findings are related to Rem2-dependent changes synaptic scaling and intrinsic excitability. Please clarify.

4) The physiological significance of the changes in mEPSC amplitudes by MD are questionable (~1 pA, ~10%). The claims need to be toned down. In comparison to this small change in amplitude, the reduction in mEPSC frequency by MD seems substantial, especially for 6 days of MD. Moreover, strikingly, Rem2 deletion affects mEPSC frequency in opposite directions for mEPSCs and mIPSCs (a decrease for the former and an increase for the latter). This could suggest a decrease in E/I balance that is a variable under homeostatic control. What is particularly puzzling is that Rem2 deletion reduced mEPSCs frequency independently of MD, but does not prevent its reduction by MD. These results may suggest that the MD-induced reduction of mEPSC frequency is independent of Rem2. These points need clarification.

5) Figure 5D, Figure 2—figure supplement 2. The observed lack of change in spine density in *Rem2^-/-^* mice upon dark rearing is not consistent with the further reduction in mEPSC frequency upon monocular deprivation, in contrast to the statement made by the authors (subsection “Rem2 is necessary for homeostatic synaptic scaling”, last paragraph). This suggests a potential presynaptic function for Rem2. While spine density analysis and whole cell recording of mini events in layer 2/3 of the V1 cortex provide some details of the defects induced by Rem2 deletion at the synapses, it is insufficient to understand what are the synaptic changes induced by MD that are affected by Rem2 removal. These points need to be clarified. Moreover, somewhat puzzling is the rather striking drop in mEPSC frequency over the 4 day period between P28 and P32 for wild type typically reared animals, and this also requires an explanation.

6) The authors should report p-values even in cases where the comparisons fall below the significance level.

# Reply: Author response

**DOI:** 10.7554/eLife.33092.021

Essential revisions:

1) The effect of Rem2 deletion and MD on intrinsic excitability of pyramidal neurons seems to be biphasic, showing a decrease 2 days after MD and an increase in the following days that re-normalizes the I/F function to the baseline. The authors cannot claim that the "neurons from Rem2^-/-^ mice failed to further increase their intrinsic excitability in response to 6 days of MD", as they do increase in comparison to 2d of MD. Furthermore, an increase in intrinsic excitability was also seen after 6 days of MD in WT mice (Figure 6). The important question is why Rem2 KO neurons show reduced activity 2d after MD, while neurons from WT mice do not. Since 2d of MD corresponds to the initial depression of visual responses in OD plasticity (Figure 3) and can also be explained by Hebbian-like long-term depression, it is not clear how this change can be stated as homeostatic. The reduction in excitability following 2d could contribute to LTD and the authors should explore this possibility experimentally. It would be important to measure spontaneous spiking activity, in addition to intrinsic excitability measurements, in order to understand the full picture.

We agree with the reviewers that the decreases in intrinsic excitability observed in the *Rem2^-/-^* mice following 2d MD, which is not observed in WT mice, raises interesting questions, that we have attempted to address below, as to how neurons respond to changes in sensory input in the presence or absence of Rem2. We also agree that we cannot conclude that this response indicates that homeostatic mechanisms are intact (or not intact with 6d MD) and have edited the text accordingly

As the reviewers suggested, we initially sought to determine if LTD was affected by Rem2 deletion in visual cortex. We attempted to induce and record LTD in acute cortical slices derived from wildtype mice for a number of months using the following experimental protocols, which we performed in consultation with members of the Bear Lab at MIT, the Shatz lab at Stanford, and the Lisman lab at Brandeis. We generated acute slices and layer 4 of the visual cortex was stimulated with either a single stimulation protocol of 900 pulses delivered at 1Hz (Jenks et al., 2017; Sidorov et al., 2015) or 900 pulses delivered at 1 Hz repeated 3 times every 25 minutes (Djurisic et al., 2013) while we recorded evoked field potentials in layer 2/3. Unfortunately, we were unable to achieve reliable LTD in our wildtype slices from visual cortex, thus we did not proceed with these experiments in *Rem2^-/-^* mice.

We therefore focused our attention on assaying the effect of *Rem2* knockout on spontaneous firing in visual cortex as the reviewers also suggested. New data in Figure 9 demonstrate that Rem2 regulates the spontaneous firing rates of layer 2/3 neurons in a cell autonomous manner.Specifically, sparse *Rem2* deletion causes a cell-autonomous increase in spontaneous firing of these neurons. Therefore, Rem2 normally functions to suppress firing, perhaps to act as a brake on increased excitability. We had hypothesized, based on the decreased intrinsic excitability observed with 2d MD in slices obtained from *Rem2^-/-^* animals, that 2d of MD would decrease spontaneous firing. However, this was not the case as 2d MD did not change the spontaneous firing rate in either control or *Rem2* deleted neurons (Figure 9). Thus, although Rem2 undoubtedly regulates both intrinsic excitability and spontaneous firing, there is not necessarily a one-to-one correspondence between these two measures in the context of short-term sensory deprivation, which presumably invokes many competing plasticity mechanisms.

We also investigated in vivo spontaneous firing rates in visual cortex in both wildtype and *Rem2^-/-^* mice at the peak (P28) and end (P34) of the critical period. New data in Figure 10 reveal a significant increase in spontaneous firing rates in *Rem2^-/-^* mice at P34 but not at the earlier time point. Thus, in the absence of Rem2, excitation is increased in cortical circuits, suggesting that Rem2 might normally function to dampen excitation. Further, the increase in spontaneous firing rate develops over a timeline that coincides with the critical period for OD plasticity, suggesting that Rem2 is required for refinement of cortical circuits.

We believe that these additional data strengthen our conclusion that Rem2 regulates cortical circuit plasticity at least in part through regulation of intrinsic properties of pyramidal neurons. Our working models is that in the absence of Rem2 the network functions at a hyper-excited state which affects the ability of the circuit to adapt to sensory deprivation through changes in synapse strength and intrinsic excitability.

2) The finding that Rem2 can regulate intrinsic excitability in a cell-autonomous manner and in the absence of sensory manipulation (Figure 7) seemingly contradicts the initial finding that Rem2 mRNA expression does not increase without sensory experience in dark-reared animals (Figure 1). This would seem to be an indication that Rem2 function is likely complicated, warranting further exploration of Rem2's role in critical period plasticity. Either tone down the claims to account for possible different interpretations, or if we are wrong, clarify how we are misunderstanding this.

Animals used for the data in Figure 7 (now Figure 8) were exposed to normal amounts of visual stimulation during their 12hr light/ 12hr dark housing. Therefore, we expect that they exhibited some ongoing expression of Rem2 that is intermediate between dark-reared animals and animals suddenly brought into the light after dark-rearing, similar to that observed in Figure 1A. We have clarified this point in the text (subsection “Rem2 functions cell-autonomously to regulate intrinsic excitability”, third paragraph).

*3)* Rem2 *KO mice display early-phase OD plasticity to a similar extent as WT mice but exhibit a deficit in late-phase OD plasticity. Specifically, upon 6 days of MD (Figure 3C), WT mice increase the ipsilateral eye response but not the contralateral eye response, while* Rem2 *KO mice increase both ipsilateral and contralateral eye responses that result in a lack of further shift in ocular dominance index in late-phase OD plasticity. Although this suggests that Rem2 normally suppresses the increase specifically of the contralateral eye responses relative to the ipsilateral eye responses, it is not obvious how these findings are related to Rem2-dependent changes synaptic scaling and intrinsic excitability. Please clarify.*

Our new data demonstrate that the spontaneous firing rate is increased at P34 in the intact cortex of *Rem2^-/-^* mice (Figure 10). Thus, the aggregate of the effect of *Rem2* KO (increased intrinsic excitability, lack of synaptic scaling, decreased mEPSC frequency) appears to be increased spontaneous activity. As the reviewers point out, the *Rem2^-/-^* animals exhibited an increase in responses for each eye during late-phase MD as measured by ISI (Figure 3C, inset). We believe that these data are consistent with our observation in vivo that the visual cortex of *Rem2^-/-^* mice is in a more excitable state compared to WT mice (Figure 10). Thus, the increased overall activity in the *Rem2^-/-^* mouse obscures any competition-dependent changes that could be observed by ISI between the ipsilateral and contralateral eyes. We have tried to clarify this point in the Results (subsection “Rem2 regulates spontaneous firing rate”, last paragraph) and Discussion.

4) The physiological significance of the changes in mEPSC amplitudes by MD are questionable (~1 pA, ~10%). The claims need to be toned down.

The observed changes of ~10% are consistent with previous reports in the literature of scaling up of mEPSC following 6d MD (Lambo and Turrigiano, 2013). We have added a line in the Results section stating that the magnitude of the effect that we observe is similar to other published studies (subsection “Rem2 is necessary for synaptic strengthening and maintenance”, third paragraph).

In comparison to this small change in amplitude, the reduction in mEPSC frequency by MD seems substantial, especially for 6 days of MD. What is particularly puzzling is that Rem2 deletion reduced mEPSCs frequency independently of MD, but does not prevent its reduction by MD. These results may suggest that the MD-induced reduction of mEPSC frequency is independent of Rem2.

Our previous studies (Ghiretti and Paradis, 2011; Moore et al., 2013) and data in the current manuscript (Figure 2—figure supplement 2, Figure 8—figure supplement 1) demonstrate that Rem2 regulates dendritic spine formation and maturation even in the absence of sensory manipulation. Thus, we believe that part of the decrease in mEPSC frequency observed upon *Rem2* KO (Figure 5D) could be due to changes in synapse density. Further, in both our study and others (Lambo and Turrigiano, 2013), mEPSC frequency does not change in neurons obtained from WT animals with either 2d or 6d MD (Figure 5D), suggesting that there is no MD-dependent regulation of mEPSC frequency per se. However, the reviewers are correct that MD further exacerbates the decrease in mEPSC frequency observed with *Rem2* KO (see Figure 5D, compare *Rem2^-/-^* TR with *Rem2^-/-^* 6d MD, p=0.008), which we agree implies the existence of other signals that regulate mEPSC frequency.

These points need clarification.

Moreover, strikingly, Rem2 deletion affects mEPSC frequency in opposite directions for mEPSCs and mIPSCs (a decrease for the former and an increase for the latter). This could suggest a decrease in E/I balance that is a variable under homeostatic control.

We take the reviewers’ point. However, because we did not directly measure E/I balance in our study, we cannot comment on this possibility.

5) Figure 5D, Figure 2—figure supplement 2. The observed lack of change in spine density in Rem2 KO mice upon dark rearing is not consistent with the further reduction in mEPSC frequency upon monocular deprivation, in contrast to the statement made by the authors (subsection “Rem2 is necessary for homeostatic synaptic scaling”, last paragraph). This suggests a potential presynaptic function for Rem2. While spine density analysis and whole cell recording of mini events in layer 2/3 of the V1 cortex provide some details of the defects induced by Rem2 deletion at the synapses, it is insufficient to understand what are the synaptic changes induced by MD that are affected by Rem2 removal. These points need to be clarified.

We agree with the reviewers’ point and have removed that line from the text. Because the mEPSC analysis in Figure 5D and the spine quantification in Figure 2—figure supplement 2 involve two different manipulations of visual experience we have moved the spine data to the beginning of the Results section describing our initial characterization of the *Rem2^-/-^* phenotype for clarity. In addition, the analysis of spine density and morphology upon sparse *Rem2* deletion (now Figure 8—figure supplement 1) during normal rearing conditions, taken together with our previously published data (Ghiretti and Paradis, 2011; Moore et al., 2013) strongly suggest that regulation of spine development is a cell-autonomous function of Rem2 signaling. We have attempted to weave together all these points in our revised manuscript.

We also agree with the reviewers that the observed decrease in mEPSC frequency over the course of the critical period and in response to monocular deprivation in *Rem2^-/-^* mice suggest that other mechanisms are in play, particularly on the presynaptic side. This is a possibility that we bring up both in the Results section and in the Discussion and that we hope to explore in the future.

Moreover, somewhat puzzling is the rather striking drop in mEPSC frequency over the 4 day period between P28 and P32 for wild type typically reared animals, and this also requires an explanation.

For the data presented in Figure 5, we performed the in vitro layer 2/3 pyramidal recordings using littermate controls in every condition to reduce inter-animal variability. For example, WT or *Rem2^-/-^* littermates at P26 were either TR or subjected to 2d MD and then sacrificed at P28 (for a total of 4 conditions) and voltage-clamp recordings were performed. Over the course of months, the P28 experiments were completed, and then the P32 experiments began (i.e. WT or *Rem2^-/-^* littermates at P26 were either TR or subjected to 6d MD and then sacrificed at P32 for recordings). Because these experiments were performed on different cohorts of animals bred over the course of months, we chose not to make comparisons or interpret differences between wildtype and *Rem2^-/-^* mice of different ages (P28 vs. P32), because we don’t believe that our data collection lends itself to this longitudinal comparison.

Nonetheless, in response to the reviewer’s question, we ran a two-way ANOVA with Tukey post hoc comparing mEPSC frequency in typically reared WT P28 and typically reared WT P32 and found a significant decrease (p=0.021, comparing WT P28 TR with WT P32 TR mEPSC frequency). In looking into the literature, it appears that changes in mEPSC frequency during this specific timeframe have not been thoroughly explored. A handful of studies have addressed changes in mEPSC frequency up to P28 in visual cortex (Desai et al., 2002; Gao et al., 2010; Picard et al., 2014), where a developmental increase in mEPSC frequency was observed. One study found a slight increase in mEPSC frequency from P23 to P38 (Goel and Lee, 2007). In addition to mEPSC frequency, morphological spine studies suggest little change in spine density during this time period (Konur et al., 2003) while other show a slight decrease (Valverde, 1971). Given that this finding has not been extensively explored in the literature, the different cohorts of animals, and the scope of this paper, we have chosen not to focus on this comparison in the text.

6) The authors should report p-values even in cases where the comparisons fall below the significance level.

We now report specific p-values throughout the text.
